# Social contact patterns in South Korea: an analysis of a survey conducted in 2023-2024

**DOI:** 10.1186/s12879-025-10706-y

**Published:** 2025-03-01

**Authors:** Woo-Sik Son, Min-Kyung Chae, Dong-Uk Hwang, Kyeongah Nah, Minsoo Kim, Jong-Hoon Kim, Jonggul Lee

**Affiliations:** 1 Research Team for Transmission Dynamics of Infectious Diseases, National Institute for Mathematical Sciences, 70, Yuseong-daero 1689 beon-gil, Yuseong-gu, 34047 Daejeon Republic of Korea; 2 Busan Center for Medical Mathematics, National Institute for Mathematical Sciences, 179, Gudeok-ro, Seo-gu, 49241 Busan Republic of Korea; 3 Epidemiology, Public Health, Impact, International Vaccine Institute, 1 Gwanak-ro, Gwanak-gu, Seoul, 08826 Republic of Korea

**Keywords:** Close contact transmission, Social contact, Contact survey, Contact matrix, Infectious disease

## Abstract

**Background:**

Understanding social contact patterns is fundamental to the study of infectious disease transmission. However, in South Korea, detailed social contact data have not been publicly available. While global research on social contact patterns has expanded, there remains a critical need for more context-specific data in South Korea.

**Methods:**

We conducted a social contact survey over two distinct weeks covering various time periods, including school vacations and national holidays. Participants provided details such as the location, duration, frequency, and type of close contact, as well as information on the contact person’s age, sex, residential area and relationship with the participant. We analyzed the data using summary statistics and the Bayesian linear mixed model.

**Results:**

A total of 1,987 participants recorded 133,776 contacts over two weeks, averaging 4.81 contacts per participant per day. The average number of contacts per day varied by age, household size, and time period. Contacts were highest in the age group 5-19, lowest in the age group 20-29, and then gradually increased up to the age group 70+. Contacts also increased with household size. Weekdays during the school semester showed the highest number of contacts, followed by weekdays during vacations, the Lunar New Year holidays, and weekends. Contact patterns differed notably by period; during the Lunar New Year holidays, closed contacts with extended family members and, therefore, subnational social mixing were enhanced.

**Conclusion:**

Our analyses across different time periods revealed significant and some unique variations of social contact patterns in South Korea. These findings can improve our understanding of infectious disease transmission in South Korea and will be useful for tailoring regional epidemiological models.

**Supplementary Information:**

The online version contains supplementary material available at 10.1186/s12879-025-10706-y.

## Background

Social contact patterns have changed dramatically during the COVID-19 pandemic, depending on social distancing measures or the degree of transmission in the community. In countries with lockdowns or nighttime curfews, the number of contacts sharply declined by approximately 28% to 88% following stringent non-pharmaceutical interventions [1–8]. Contact patterns also shifted due to behavioral changes, such as wearing masks, refraining from physical contact, or canceling leisure activities [2, 3]. In South Korea, special prevention policies (e.g., stay-at-home campaigns, highway screening clinics) were implemented in February 2020 due to concerns about the spread of SARS-CoV-2 during the Lunar New Year holidays. By the end of the month, the non-commuting mobility of population had dropped by 25% compared to the previous year [9]. Recently, as the COVID-19 pandemic has eased, contact patterns have shown signs of returning to pre-pandemic levels [10].

Understanding contact patterns is critical for accurately modeling the spread of infectious diseases. Accounting for heterogeneity in contact patterns across groups (e.g., age, sex, location) enhances the accuracy of infectious disease forecasts and the evaluation of the effectiveness of different mitigation strategies. For example, children often have higher contact rates, making them significant drivers of many respiratory infections [11, 12]. Temporal variations in contact patterns, such as differences between weekdays and weekends or during holidays, can also significantly alter disease trajectories. Ewing et al. (2017) demonstrated that during holiday periods, local transmission may decrease due to school closures while long-distance transmission increases due to increased travel activity, either delaying or advancing epidemic peaks, depending on timing [13]. These findings underscore the importance of capturing temporal and demographic variations in contact patterns to refine epidemiological models and inform public health interventions. During the COVID-19 pandemic, several studies have estimated changes in contact patterns in response to rapidly changing epidemic conditions and evaluated in real time the impact of mitigation policies in South Korea [14–18]. However, the contact matrices utilized in these studies were adapted from the POLYMOD study [12, 19] due to the absence of a representative contact matrix for South Korea.

This study investigates the contact patterns in South Korea across various periods, including weekdays during the semester, weekdays during the vacation, weekends, and Lunar New Year holidays. The survey was conducted in two one-week long periods during the winter season of 2023-24, covering a total of 1,987 Korean persons. The study offers critical data for refining epidemiological models and enhancing public health strategies by highlighting and quantifying contact matrices over different periods in South Korea.

## Methods

### Survey design & data collection

The survey was conducted during two separate weeks: (a) Dec. 6 to 12, 2023, and (b) Feb. 7 to 13, 2024. The latter period includes the Lunar New Year holidays and the winter vacation for students and teachers. Participants were recruited through a quota sampling method to ensure their age group and regional area matched the actual population distribution of South Korea. The age groups were categorized into 8 segments (0–9, 10–19, 20–29, 30–39, 40–49, 50–59, 60–69, 70–79 years), and residential areas were classified into 5 major regions in South Korea. For detailed information about these regions (Metropolitan, Chungcheong, Gangwon, Youngnam, and Honam), please refer to the interactive webpage described in Age-grouped contact matrix section. For the comparison between sample and population distributions of age and region, see Figure S2. All 1,987 participants recorded their daily social contacts for two weeks. The social contacts of the participants were recorded using two different methods based on their age: the age groups 0–18 and 65+ used a paper diary, while the age group 19–64 used a mobile web page. This approach leveraged the online panel available for the age group 19–64. For children 14 years old and younger, a parent or guardian assisted in recording their contacts. Additionally, all participants received help from a trained staff member in filling out either their paper diary or mobile web page.

We used the same definition of a close contact as in the previous study Ref. [12]. A close contact is defined either as a physical contact involving skin-to-skin interaction, or as a non-physical contact characterized by an interactive dialogue consisting of three or more words without skin contact. Participants reported details about each contact, including the age, sex, and residential area of the contact person, as well as the relationship with the contact person, and the location, duration, frequency, and type (physical or non-physical) of the interaction. All participants provided their age, sex, residential area, occupation, and the size and age of any cohabiting members, whether they were family members or not.

### Data analysis

We assessed the variations of the number of close contacts per day (i.e., daily contact rate) by age group while controlling for various epidemiologically important variables: sex, household size, and date. These variables were selected based on insights from previous studies [10–12, 19]. While age is discrete and available in years, it was grouped to reflect the epidemiological importance of certain age groups (e.g., 0–4 years old) and school-age children and young adults (e.g., 5–19 years old) [12] which could influence the number of contacts. Participants in households of size six or more members were merged with those in household of size five, creating the category of “5+”, due to the small number of participants in larger households. The survey dates were categorized into four distinct periods: weekdays during the semester (Period I), weekdays during vacation (Period II), weekends (Period III), and Lunar New Year holidays (Period IV), as we hypothesized that daily contact rates would vary across these periods. In addition, we analyzed the subset of the data representing a typical week (i.e., excluding vacation and Lunar New Year holidays) in which date is represented by the day of the week (see Supplementary Information).

We used the Bayesian generalized linear mixed model to account for the repeated measures of daily contact rates (i.e., 14 measures per participant). Full posterior distributions as well as prior distributions of the parameters appear in Supplementary Information. We chose a negative binomial distribution to capture that the daily number of contacts across participants is overdispersed (variance-to-mean ratio > 3). In particular, the daily number of close contacts, *y* was modeled as a random number distributed as negative binomial distribution with mean, $\mu$, and dispersion size (i.e., dispersion parameter), $\phi$, $\textrm{NegBinomial}(\mu ,\phi )$. Expectation and variance of the distribution are given as $\mu$ and $\mu + \frac{\mu ^2}{\phi }$ and the $\textrm{NegBinomial}(\mu ,\phi )$ converges to $\textrm{Poisson}(\mu )$ if $\phi \xrightarrow \infty$.

1 $$\begin{aligned} \textrm{Pr}(\textrm{Y} = y_i|\theta ) & = \textrm{NegBinomial} ( y_i|\mu ,\phi ) = \left( \begin{matrix} y_i+\phi -1 \\ y_i \end{matrix} \right) \left( \frac{\mu }{\mu +\phi } \right) ^ {y_i} \left( \frac{\phi }{\mu +\phi } \right) ^\phi \end{aligned}$$

2 $$\begin{aligned} \textrm{effect}_i & \sim \mathcal {N} (0, \tau )\end{aligned}$$

3 $$\begin{aligned} y_i & \sim \textrm{NegBinomial}(\mu _i, \phi )\end{aligned}$$

4 $$\begin{aligned} \textrm{log}(\mu _i) & = \alpha + \beta _{\textrm{age}}\textrm{age}_i + \beta _{\textrm{hh}}\textrm{householdsize}_i + \beta _{\textrm{date}} t + \beta _{\textrm{sex}}\textrm{sex}_i + \textrm{effect}_i \end{aligned}$$

Models incorporating different covariate combinations were fitted, and their relative performance was assessed using the expected log predictive density (elpd) from leave-one-out (LOO) cross-validation [20] and the Watanabe-Akaike information criterion (WAIC) [21]. The final model includes age, sex, date, and household size as covariates, although the model excluding sex was preferred according to the WAIC and LOO cross-validation. This decision was based on the minimal difference in performance between the two models, their similar parameter estimates (e.g., see Fig. S6 for the dispersion parameter, $\phi$), and the relevance of sex in understanding social contact patterns. Details on the elpd values are provided in the Supplementary Information (Table S1 in Section 1.2.3). Bayesian model fitting was performed using the ‘brms’ package [22] in R, which utilizes the probabilistic programming language Stan [23]. Four independent Markov chains were run for 20,000 iterations, with the first 10,000 iterations used for warmup. The remaining 10,000 iterations from each of the four chains were used for inference. Convergence was confirmed by examining the trace plots and ensuring the $\hat{R}$ diagnostic being less than or equal to 1.01.

To derive the age-grouped contact matrix, we accounted for reciprocity following the method described in the previous study [24]. Initially, the contact matrix without correction is calculated as $m_{ij} = x_{ij}/g_{j}$, where $x_{ij}$ represents the total number of contacts between participants in age group *j* and contact people in age group *i*, and $g_j$ is the number of participants in age group *j*. Considering the reciprocity, the total number of contacts at the population level should be equal, which means $m_{ij} G_j = m_{ji} G_i$, where $G_j$ is the size of the population in age group *j*. The adjusted age-grouped contact matrix considering reciprocity is then expressed as $m_{ij}^{C}=C_{ij}/G_{j}$. Here, $C_{ij}=(m_{ij} G_{j} + m_{ji} G_{i})/2$ represents the average number of contacts in age group *i* as reported by a participant in age group *j*. Finally, the mean and confidence interval of age-grouped contact matrix is derived through bootstrapping.

## Results

### Characteristics of participants

Among the 2,415 participants in the preliminary survey, a total of 1,987 participated in both the first and second rounds of the survey. Of these, 1,364 (68.7%) participated through a mobile web page (the age group 19–64), while the remaining 613 (31.3%) participated via a paper diary. Among the participants who took part in the paper diary, there were 316 participants (15.9%) in the age group 0–18 and 307 participants (15.5%) in the age group 65+. The largest number of participants in any age group was in the age group 40–49, with 352 participants (17.7%). The age distribution by group was similar to that of the South Korean population in 10-year age increments, but the largest proportion in the population distribution was in the age group 50–59 (17.5%) in Supplementary Information (Section 1.2) [25]. Approximately 57.6% of participants reported living in households with 3–4 members. About 47.9% of participants resided in Seoul and Gyeonggi Province, while Sejong and Jeju each had only about 20 participants. The occupation with the largest proportion among participants was office/technical work (33.1%), followed by students, including university/graduate students (16.5%), and full-time homemakers (13.5%).

### Number of contacts

A total of 133,776 contacts were recorded for 1,987 participants over a period of 14 days, leading to a mean of 4.81 contacts per participant per day. The contact rate (i.e., the number of contacts per day) was right skewed and variance (16.57) was over 3 times larger than the mean (4.81). Estimates from the Bayesian generalized linear mixed model generally agree with the crude estimates and 95% credible intervals encapsulate crude estimates (Table 1). Full posterior distributions for the parameters as well as prior distributions appear in Supplementary Information (Section 1.2.2). The contact rate varies across age, household size, and period. Regarding the effect of age, the contact rate was highest for school-age children and adolescents (i.e., 5–19 years old) and lowest among 20–29 years old. The contact rate increased again in older age groups (> 60 years old), reaching levels similar to those observed in 0–4 years old. Household size also influenced the contact rate, with each additional household member being associated with an 8–20% increase in contacts. The contact rate was highest on weekdays during the semester (Period I), with a gradual decline in the order of weekdays during the vacation (Period II), Lunar New Year holidays (Period IV), and weekends (Period III).

**Table 1:** Summary estimates of social contacts by age group, sex, period, and household size

Category	Covariates	No. part^a^	Mar mean (sd)^b^	CR^c^	Est. (95% CI)^d^
Age group	0–4	60	5.67 (2.30)	1.00	1.00
	5–9	84	8.53 (3.84)	1.50	1.43 (1.21 - 1.69)
	10–14	96	8.33 (3.48)	1.50	1.38 (1.16 - 1.63)
	15–19	83	7.83 (3.41)	1.40	1.28 (1.08 - 1.52)
	20–29	256	3.22 (1.95)	0.57	0.52 (0.45 - 0.61)
	30–39	292	3.50 (1.83)	0.62	0.62 (0.53 - 0.72)
	40–49	352	4.09 (1.90)	0.72	0.70 (0.60 - 0.81)
	50–59	316	4.42 (2.18)	0.78	0.75 (0.65 - 0.87)
	60–69	273	5.23 (3.31)	0.92	0.91 (0.78 - 1.06)
	70+	166	5.49 (3.24)	0.97	1.04 (0.88 - 1.23)
Sex	Female	1036	4.83 (2.90)	1.00	1.00
	Male	951	4.78 (3.09)	0.99	0.94 (0.90 - 0.99)
Period	Weekdays during the semester (I)	1987	5.49 (4.60)	1.00	1.00
	Weekdays during the vacation (II)	1987	4.90 (3.92)	0.89	0.89 (0.88 - 0.91)
	Weekends (III)	1987	3.67 (3.09)	0.67	0.68 (0.66 - 0.69)
	Lunar New Year holidays (IV)	1987	4.46 (3.72)	0.81	0.82 (0.80 - 0.83)
HH size ^e^	1	254	3.50 (2.36)	1.00	1.00
	2	453	4.54 (2.89)	1.30	1.21 (1.11 - 1.31)
	3	583	4.86 (2.70)	1.40	1.31 (1.21 - 1.42)
	4	561	5.25 (3.17)	1.50	1.49 (1.38 - 1.62)
	5+	136	6.13 (3.75)	1.80	1.64 (1.47 - 1.83)

^a^No. part = number of participants

^b^Mar mean (sd) = marginal mean with standard deviation

^c^CR = crude ratio

^d^Est. = estimates with 95% credible interval

^e^HH size = household size

**Fig. 1:**
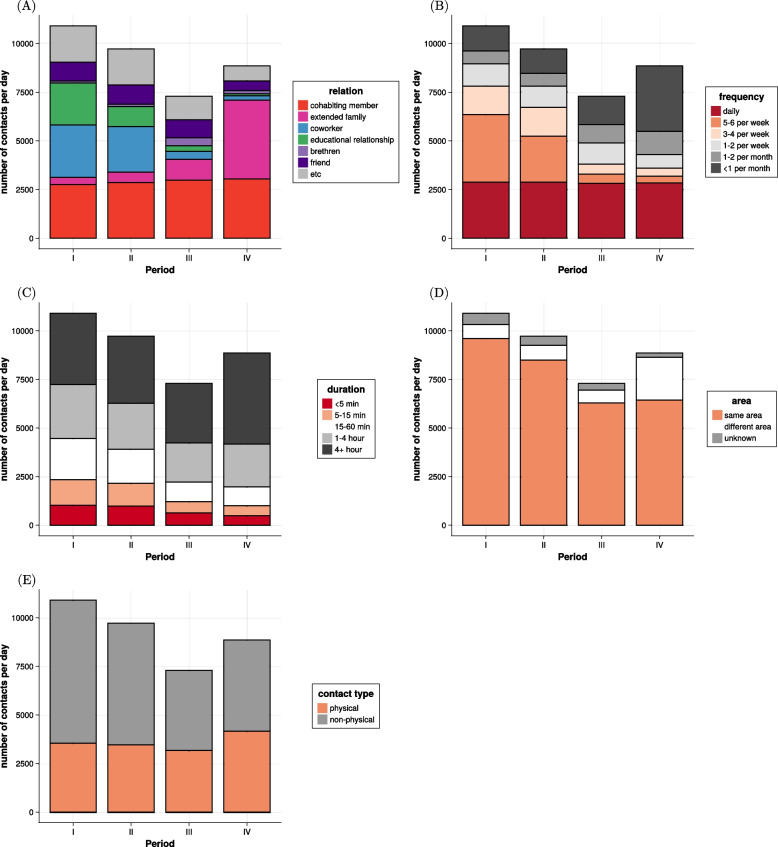
The stacked bar chart for the number of contacts per day in each period by **A** relation, **B** frequency, **C** duration, **D** residential area, and **E** contact type

We analyzed the variation in social contacts across periods, as shown in Fig. 1, which shows stacked bar charts for the number of contacts per day categorized by (A) relation, (B) frequency, (C) duration, (D) residential area, and (E) contact type. In terms of relation with the contact (Fig. 1A and Table S3), the number of contacts with the cohabiting members remains almost the same in all periods. However, in Period II the number of close contacts with the educational relationship falls to 51% of the level in Period I, showing the impact of the school vacation. In Period III, due to the weekend, contacts with coworkers and educational relationships decrease by more than 80%, while contacts with extended family (such as grandparents, uncles, aunts, grandchildren, etc.) and brethren increase by more than 200%. In Period IV, contacts with extended family increase by more than 1000%, indicating increased social mixing during Lunar New Year holidays. Regarding the frequency of contacts (Fig. 1B and Table S4), daily contacts remain constant across all periods, which corresponds to contacts with the cohabiting members. In Periods II and III, contacts 3–6 per week decrease compared to those in Period I. In Period IV, contacts less frequent than once a month increase by more than 160%, representing to contacts with extended family. The results on contact duration given in Fig. 1C and Table S5 show that all contact durations decrease compared to those in Period I, except for the contact duration of more than 4 hours in Period IV. This corresponds to contact with cohabiting members and extended family during Lunar New Year holidays. Figure 1D and Table S6 show that contacts with people living in different residential areas increase in Period IV. This implies an enhancement in subnational social mixing during Lunar New Year holidays. Finally, the results on the type of contacts are given in Fig. 1E and Table S7. It shows that physical contacts increase during Lunar New Year holidays.

**Fig. 2:**
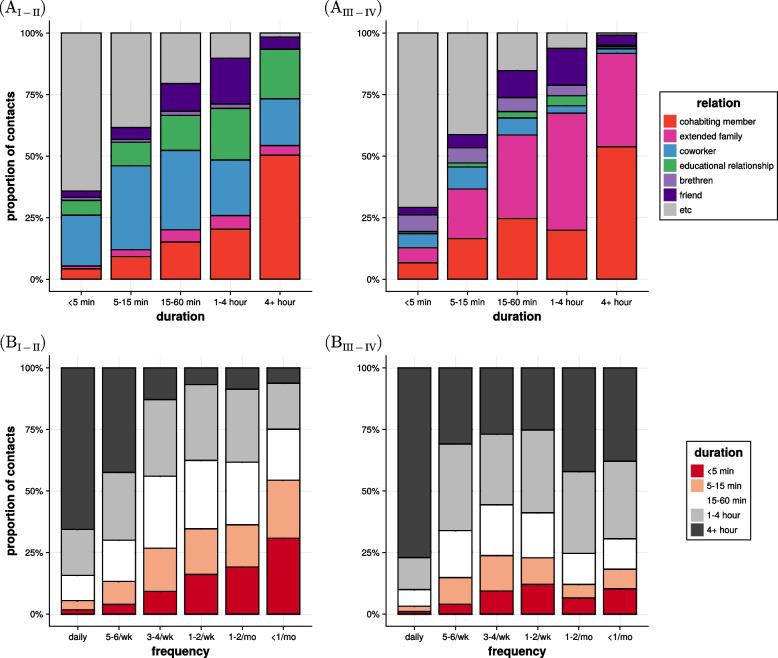
The proportion of social contacts which shows the correlation between (A) duration and relation and (B) frequency and duration. Subfigures (I-II) and (III-IV) show the proportion of contacts in Periods I-II, and III-IV, respectively

We also analyzed the correlations between the characteristics of close contacts for each period. We found that Periods I and II, as well as Periods III and IV, have the same pattern. Therefore, we organized the correlation analysis into two groups: Periods I-II and III-IV. Figure 2(A_I-II_) and (A_III-IV_) show the correlation between the duration and relation of close contacts in Periods I-II and III-IV, respectively. In all periods, the proportion of contacts with cohabiting members and extended family increases as the duration of contacts increases. In Periods I and II, for close contacts lasting more than 4 hours, the proportion with cohabiting members and extended family remains below 60%. However, in Periods III and IV, it exceeds 90%. In Fig. 2(B_I-II_) and (B_III-IV_), we illustrated the correlation between the frequency and duration of close contacts. In Periods I and II, the duration of close contacts decreases with decreasing frequency, which is consistent with the results presented in Fig. 1D of Ref. [12]. However, in Periods III and IV, although the frequency of contacts decreases, the proportion of contacts lasting more than 4 hours does not decrease and remains constant. Unlike the results of Fig. 2, where all correlations reveal different patterns over the periods, the correlations between relation versus contact type and duration versus contact type are consistent across all periods (see Fig. S9).

### Age-grouped contact matrix

**Fig. 3:**
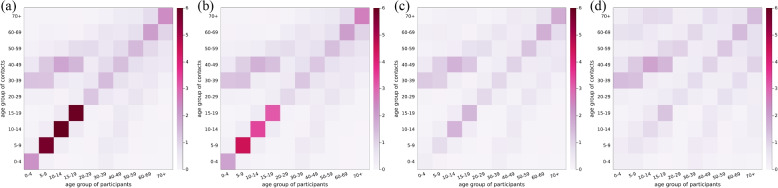
The age-grouped contact matrix for each period. The contact matrices are the average daily contact number: **a** weekdays during the semester (Period I), **b** weekdays during the vacation (Period II), **c** weekends (Period III), and **d** Lunar New Year holidays (Period IV)

**Fig. 4:**
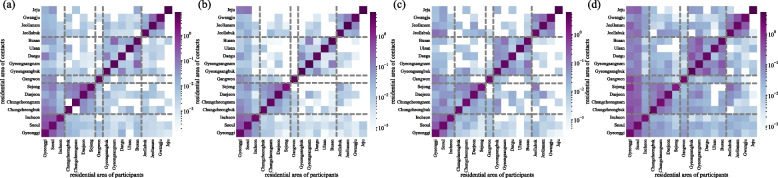
The contact matrix of residential area for each period. The contact matrices are the average daily contact number: **a** weekdays during the semester (Period I), **b** weekdays during the vacation (Period II), **c** weekends (Period III), and **d** Lunar New Year holidays (Period IV). South Korea is divided into 17 provincial-level administrative regions, which we grouped into 5 major regions indicated by dashed lines, by combining neighboring regions. We used a logarithmic color scale to improve readability, as contacts between different regions are much fewer than those within the same region

**Fig. 5:**
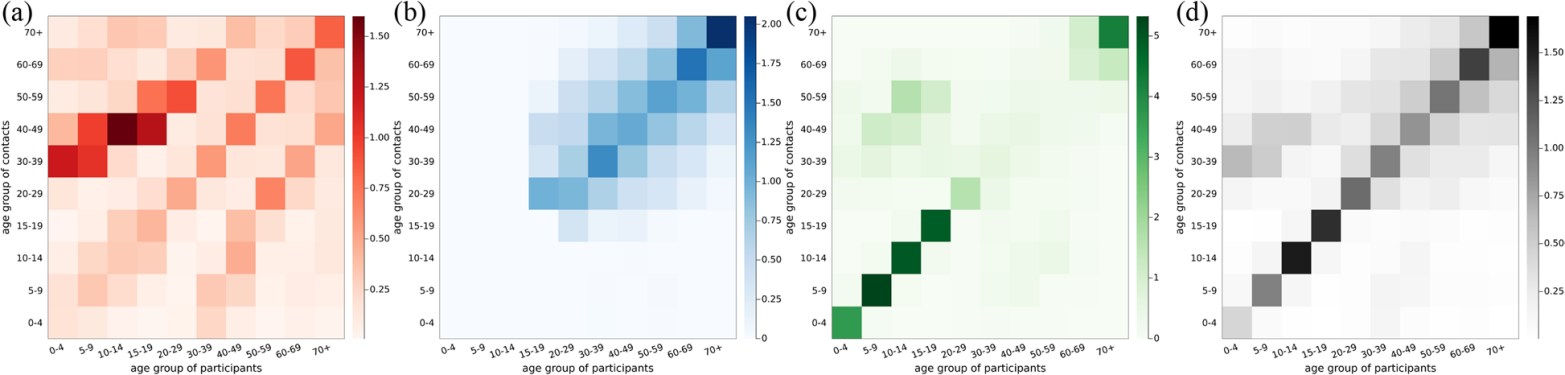
The age-grouped contact matrix by each location. The contact matrices are the average daily contact number **a** at home, **b** in the workplace, **c** in the educational facility, and **d** in other locations. Note that each color bar has a different range

We computed contact matrices from various perspectives (see Figs. 3, 4, and 5). Each contact matrix was calculated using the bootstrap method and considering reciprocity described in Methods ‘Data analysis’ section. We bootstrapped 2000 iterations. In the contact matrix, each element represents the estimated average number of contacts per participant per day. The color bar indicates the daily contact number.

Figure 3 shows the age-grouped contact matrices for when social contacts occurred. The Periods I and II have highlighted diagonal elements of the matrix compared to the Periods III and IV. It indicates that people have more contact with the same age group in Periods I and II. In Period IV, the number of contacts in off-diagonal elements increases compared to Periods I, II, and III. It shows that social mixing between different age groups is enhanced in Period IV.

Figure 4 shows the contact matrices of residential area for each period. South Korea is divided into 17 provincial-level administrative regions. We categorized the residential areas based on these 17 regions, and grouped neighboring regions into 5 major regions, shown with dashed lines in Fig. 4. It is important to note that the regions are the residential area of the participants and contact people, not the locations where the social contacts occurred. In Fig. 4, we only considered cases where the participants knew the residential area of the contact person and excluded cases where the residential area was unknown. Participants mainly had contacts with people who lived in the same region. However, contact numbers with people from different residential area increased in Period IV. We used a logarithmic color scale in Fig. 4 to improve readability, as the contact numbers in the off-diagonal elements (contacts between different regions) are much smaller than those in the diagonal elements (contacts within the same region).

Figure 5 shows the age-grouped contact matrices by location, where social contacts occurred. It should be noted that in our survey, participants could have met the same contact person in multiple locations. The contact matrix was calculated using the data specific to each location without consideration of the contact date. While the contact patterns in each location were distinct, all locations exhibited strong assortative mixing patterns-higher numbers of contacts within the same age groups-in certain age groups. Most intergenerational mixing occurred at home (see Fig. 5a). The home is a living space where many contacts occur beyond household members (e.g., with service workers such as repairmen). Note that there is strong intergenerational mixing starting from the 30–39 age group, alongside increasing assortative mixing as contacts increase with age. The contact pattern in the workplace is prominent between adult groups (see Fig. 5b). In our survey results, the most common reason for contact between adult and minor groups in the workplace was children visiting their parents’ workplaces. The highest daily numbers of contacts (diagonal elements) for the minor groups in the educational facility were 3.68, 5.33, 4.98, and 4.92, respectively, which is significantly higher compared to other locations (see Fig. 5c). The contact matrix results for other locations and other contact matrices can be found in Supplementary Information. The interactive webpage at https://observablehq.com/@nims-epid/age-grouped-contact-matrix-of-south-korea allows users to define their own configurations for age groups and time periods, and then generate the corresponding contact matrix. Additionally, users can obtain the contact matrix for residential area based on their chosen administrative region and time periods at https://observablehq.com/@nims-epid/contact-matrix-with-maps-of-residential-area-of-pa.

## Discussion

We analyzed social contact patterns using a survey conducted over two separate weeks in South Korea, covering weekdays, weekends, the semester, school vacation, and Lunar New Year holidays. Our analysis revealed distinct social contact patterns for each period, consistent with existing studies that emphasize the influence of academic schedules, daily routines, and cultural traditions. The structured nature of academic life promotes more frequent social engagement during semesters, while vacations result in less social contact due to a lack of routine and geographic dispersion. This pattern was clearly observed in our contact survey results, with the highest contact rates recorded during the semester and a noticeable decline during vacation periods [26–31]. Additionally, weekdays showed higher contact rates than weekends due to routine work or school activities [3, 26–37], while weekends were marked by more personal or family-oriented activities. During holiday periods, particularly the Lunar New Year, there was a significant increase in contact with extended family members, reflecting the cultural importance of family gatherings.

Our results provide significant insights into social contact patterns in South Korea as the COVID-19 pandemic transitions into an endemic phase. Using the next generation approach [1, 2, 10, 11, 38–40], which compares the dominant eigenvalues of contact matrices under the assumption that all age groups equally contribute to transmission, we found that the ratios of the basic reproduction numbers across the four periods ranged from 0.52 to 0.70 (Table S13). This suggests that social contacts have not yet fully returned to pre-pandemic norms, underscoring the pandemic’s lasting impact on social behaviors. Similar trends have been observed in other regions, such as the UK, Netherlands, and Belgium, where contact numbers remain below pre-pandemic levels [10, 41].

Our findings also reveal unique patterns that differ markedly from those reported in previous studies. The number of contacts with school-age children (5–19 years) was as expected, at its highest [1, 11, 12, 19, 42, 43]. However, in the 20–29 age group, the contact rates decreased sharply, and then increased with age, in contrast to earlier social contact patterns (Table S14 and Fig. S16). In addition, the age group 70+ was found to have the highest contact rates among those aged 20 and above, which is also different from the previous studies [12]. This discrepancy may be due to rapid changes in social behavior and demographic shifts. With the spread of telework and online classes since the pandemic, younger adults aged 20–29 are increasingly engaging in non-face-to-face (online) interactions rather than face-to-face interactions. Meanwhile, in South Korea, the elderly population is rapidly growing along with their participation in socio-economic activities. In particular, the expansion of “Caring for Grandchildren”, in which grandparents care for their grandchildren, and increased elder community activities likely contribute to this trend [44–46]. Countries experiencing similar demographic transitions observe comparable shifts in intergenerational mixing and social behaviors. These factors may explain why social contact patterns differ from previous studies; however, further sociological research is needed to clarify these relationships.

These discrepancies suggest that mathematical models of infectious disease transmission relying on pre-pandemic contact matrices may no longer provide accurate predictions for South Korea during the COVID-19 pandemic and beyond. Several studies have used the old contact matrix projected from the POLYMOD study in their models for COVID-19 in South Korea, which may not fully account for the reduced social interactions among young adults or the unexpectedly high contact rates among the elderly. This could lead to errors in estimating the basic or effective reproduction number and forecasting disease spread, potentially resulting in sub-optimal public health interventions. Updating contact matrices to reflect current social behaviors and demographic trends is essential. This can be achieved by conducting regular and localized contact surveys that capture evolving changes in social interactions, as demonstrated by the CoMix study in the EU [2, 3, 47]. Moreover, models could be improved by accommodating varying contact patterns across different periods, such as weekdays versus weekends and holiday seasons [48].

While this study provides valuable insights, several limitations should be acknowledged. The extended 14-day reporting period may have led to reporting fatigue and potential underreporting, thereby introducing bias and reducing the accuracy of contact intensity estimates over time [49–51]. To address these issues, future studies could incorporate statistical methods to adjust for biases caused by reporting fatigue [51]. Additionally, our study had excluded the foreign population, which may affect how widely the findings can be generalized. The absence of data on participants’ health conditions, mask use, and other behavioral factors further limits the ability to assess transmission dynamics in special subsets of populations separately [27, 32, 52–56]. Despite these limitations, this study offers a foundational dataset for understanding social contact patterns in South Korea, which can serve as a critical resource for refining epidemiological models and informing public health strategies.

## Conclusion

This study offers the first comprehensive analysis of social contact patterns in South Korea across various periods, including weekdays, weekends, school vacations, and Lunar New Year holidays. Contact rates were highest during weekdays in the semester and lowest during weekends, with Lunar New Year holidays enhancing inter-regional mixing and family interactions. The post-pandemic shifts in behavior, including reduced contacts among young adults and increased interactions among older adults, underscore the importance of incorporating temporal and demographic heterogeneity into epidemiological models to improve predictions and public health strategies. These findings highlight the impact of cultural and temporal factors on contact patterns, which can inform tailored interventions in other countries with similar social structures or holiday-driven mobility.

## Supplementary Information

Supplementary Material 1. Details on statistical analysis, including survey design, contact numbers, age-grouped contact matrix, and comparison of contact patterns between pre-pandemic and pandemic levels.

## Data Availability

The interactive webpage in the Results section provides access to both the age-grouped contact matrix and the contact matrix for residential areas. The datasets used in this study are available upon reasonable request from the corresponding author.
