# Supplementary material for: Social contact patterns in South Korea: an analysis of a survey conducted in 2023-2024
Source: BMC Infect Dis. 2025 Mar 1;25:295. doi: 10.1186/s12879-025-10706-y (PMC11871801; doi:10.1186/s12879-025-10706-y)
Supplement: Supplementary file 1 — Supplementary Material 1. Details on statistical analysis, including survey design, contact numbers, age-grouped contact matrix, and comparison of contact patterns between pre-pandemic and pandemic levels. [file 12879_2025_10706_MOESM1_ESM.zip › survey_SI.pdf]

Supplementary Information

Contents

1 Methods 2
1.1 Survey design 2
1.2 Participants 2
1.3 Bayesian linear mixed model 3
1.3.1 Prior distribution 3
1.3.2 Posterior distribution 3
1.3.3 Variable selection and model comparison 3

2 Results 9
2.1 Number of contacts 9
2.2 Age-grouped contact matrix 15
2.3 Contact patterns compared to the pre-pandemic and pandemic level 23

1 Methods

1.1 Survey design

Mon	Tue	Wed	Thu	Fri	Sat	Sun
Dec.4 .2023	5	6	7	8	9	10
11	12	13	14	15	16	17

Period I
Period III

Mon	Tue	Wed	Thu	Fri	Sat	Sun
Feb.5 .2024	6	7	8	9	10	11
12	13	14	15	16	17	18

Period II
Period IV

Figure S1: Survey period on the calendar. The time frame of the survey is divided into four periods: (I) weekdays during the semester, (II) weekdays during the vacation, (III) weekends, and (IV) Lunar New Year holidays. Each color represents each period.

The 1,987 participants surveyed, representing a demographically matched population in region and age group, during two separate weeks: Round 1 (Dec. 6-12, 2023) and Round 2 (Feb. 7-13, 2024). Fig. S1 shows the survey period on a calendar. The time frame of the survey is divided into four periods: weekdays during the semester (Dec. 6-8 and 11-12, 2023), weekdays during the vacation (Feb. 7-8 and 13, 2024), weekends (Dec. 9-10, 2023), and Lunar New Year holidays (Feb. 9-12, 2024). Each period is labeled Periods I, II, III, and IV.

1.2 Participants

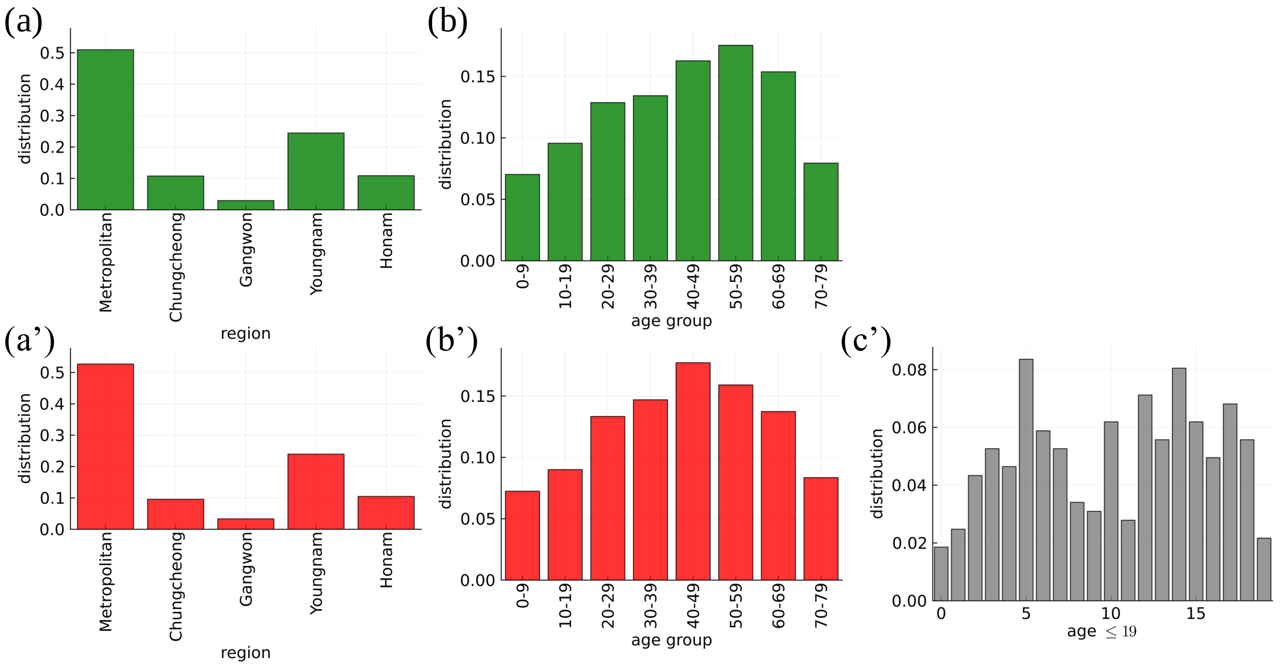

Figure S2: (a, b) The whole population's data in South Korea. (a'-c') Survey participants' data. (a, a') Population distribution by residential area. (b, b') Age group distribution. (c') Age distribution under 20-year-old.

Participants were recruited using a quota sampling method to match the distribution of the population by region-based age group in South Korea. In Fig. S2, the upper panel shows the total distribution of South Korea's population, and the lower panel shows the distribution of survey participants. Figs. S2(a) and (a') show the population distributions by residential area. We are grouped the entire region into five major areas. Figs. S2(b) and (b') show the age group distributions with a 10-year interval. The graphs have the same pattern, indicating that our participants are representative of the Korean population. Our survey also allowed infants to participate. Fig. S2(c') shows the age distribution of participants aged 19 and under, with a 1-year interval.

1.3 Bayesian linear mixed model

1.3.1 Prior distribution

We chose weakly informative prior distributions for the parameters of interest. That is, prior distributions were chosen to influence the results as little as possible while providing at least some regularization to considerably improve convergence and sampling efficiency [1].

$$
\begin{aligned}
\alpha &\sim \text{Normal}(0, 5, 0, \infty) \\
\beta &\sim \text{Normal}(0, 2, 0, \infty) \\
\sigma &\sim \text{Normal}(0, 2, 0, \infty) \\
\phi &\sim \text{InverseGgamma}(0, 2)
\end{aligned}
$$

1.3.2 Posterior distribution

We graphically present the prior and posterior distributions of the parameters for the relative contact rate by age, date, household size, and sex.

1.3.3 Variable selection and model comparison

We fitted several models that include different combinations of covariates or assume a different distribution for the outcome (negative binomial vs. poisson). The final model that includes age, date, sex, and household size as covariates was chosen based on the Watanabe-Akaike Information Criterion (WAIC), Leave-One-Out (LOO) cross-validation, and also epidemiological importance of the covariates. In particular, as seen in Table S1, three models (Model ID 1-3) are similar in terms of elpd compared to the rest of the models. Although the simpler models that does not include sex (Model 1) can be selected according to LOO cross-validation or WAIC, we chose the model that includes sex (Model 3) as the final model because seeing the impact of sex on contact would be epidemiologically important. WAIC and LOO metrics were computed using the 'loo_compare' function of the 'brms' [1] and 'loo' packages [2].

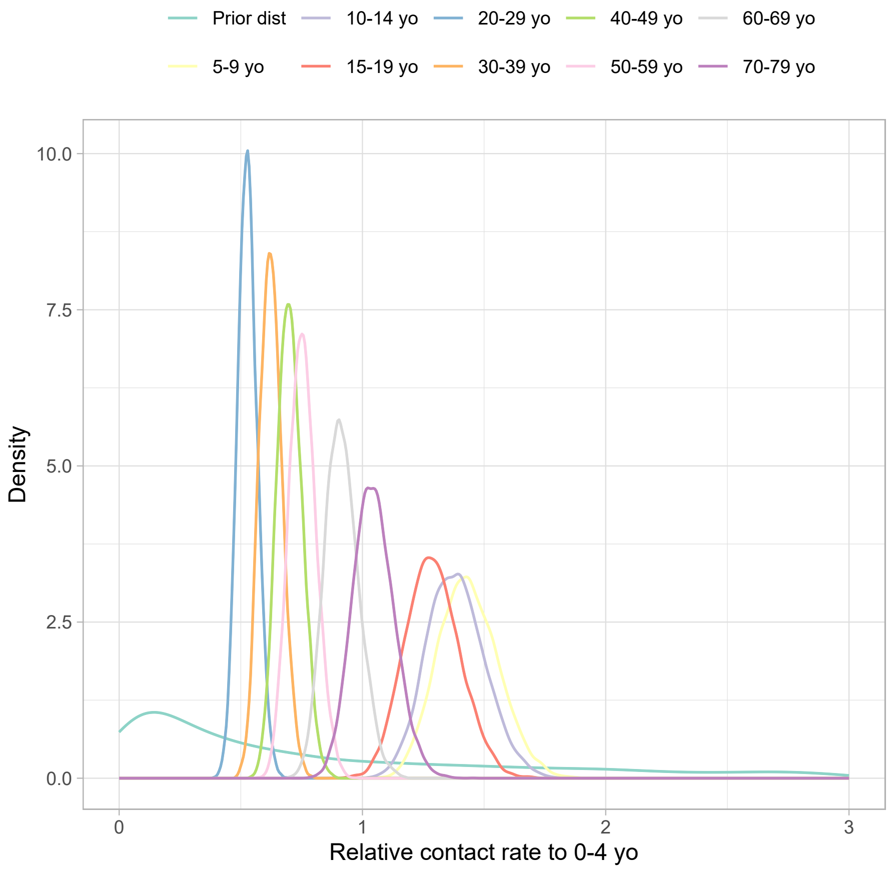

Figure S3: Inference for β_{age} parameter along with the prior distribution.

Table S1: Model comparison

**Model ID**	**Distribution**	**Covariate**					**LOO**		**WAIC**	
		age	sex	date	household size	id	elpd_diff	se_diff	elpd_diff	se_diff
1	NegBin	O	X	O	O	O	0	0	0	0
2	NegBin	O	O	O	X	O	-2.6981	3.8843	-1.1557	3.8225
3	NegBin	O	O	O	O	O	-4.2737	1.7052	-1.3682	1.2207
4	NegBin	O	X	X	X	O	-745.59	45.255	-745.17	45.233
5	NegBin	O	X	O	X	O	-747.19	44.866	-746.20	44.847
6	NegBin	O	X	X	O	O	-747.19	44.866	-746.20	44.847
7	NegBin	O	O	X	X	O	-747.64	45.249	-747.20	45.201
8	NegBin	X	X	X	O	O	-764.25	45.488	-762.30	45.477
9	Pois	O	O	O	O	O	-1036.3	88.462	-1033.8	88.672
10	NegBin	O	O	O	O	X	-6734.0	129.62	-6745.4	129.52
11	NegBin	O	X	O	O	X	-6739.9	129.57	-6751.3	129.47
12	NegBin	O	X	X	O	X	-7179.1	134.43	-7190.5	134.33
13	NegBin	O	X	X	X	X	-7179.1	134.43	-7190.5	134.33

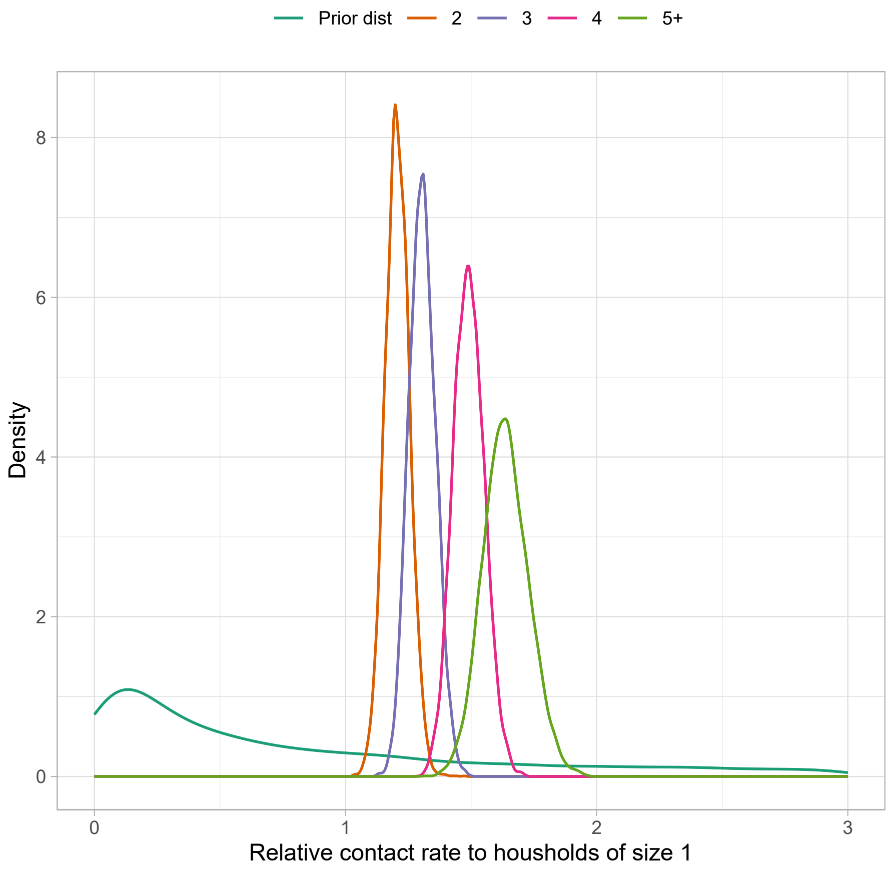

Figure S4: Inference for β_{hh} parameter along with the prior distribution.

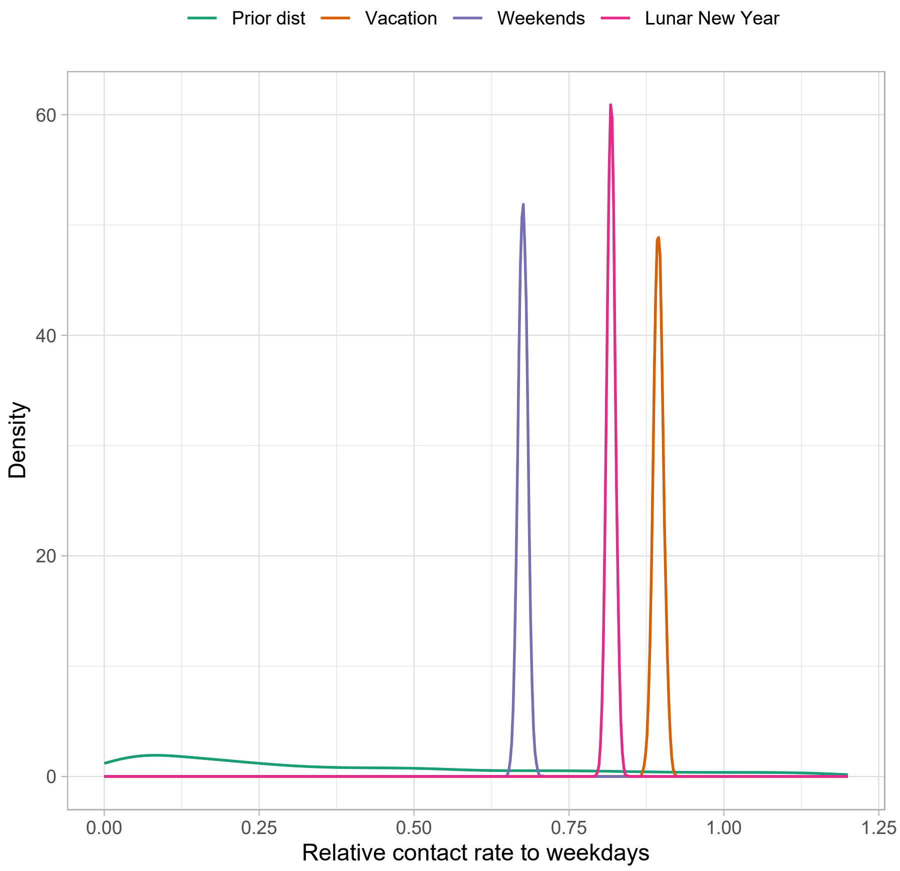

Figure S5: Inference for β_{date} parameter along with the prior distribution.

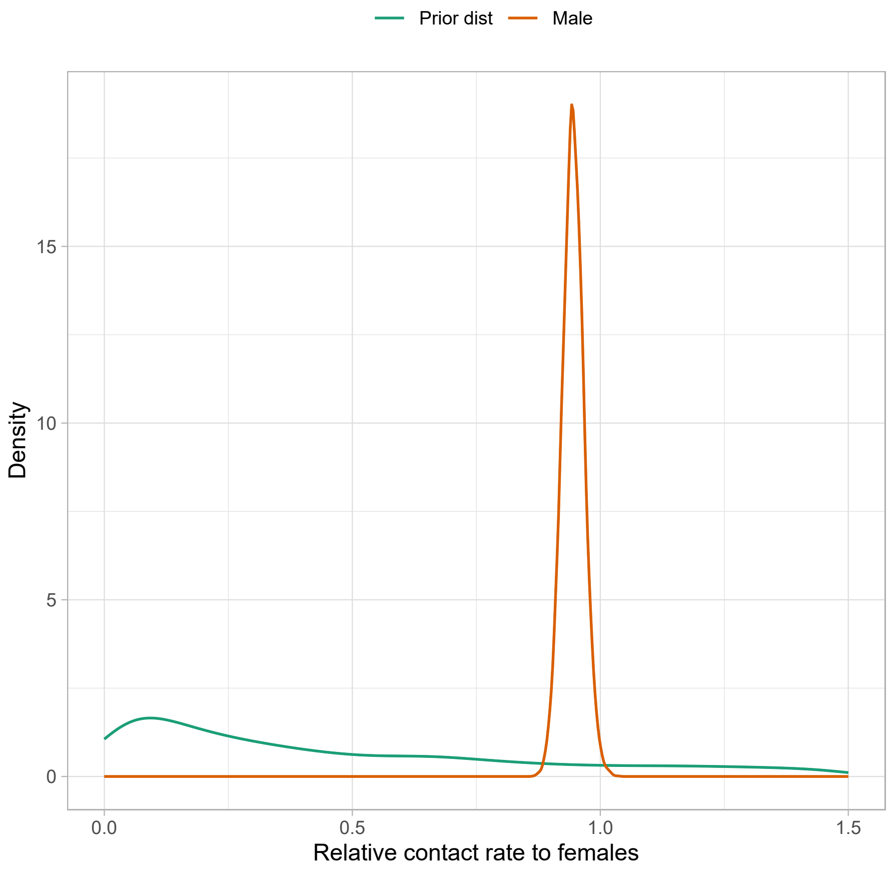

Figure S6: Inference for β_{sex} parameter along with the prior distribution.

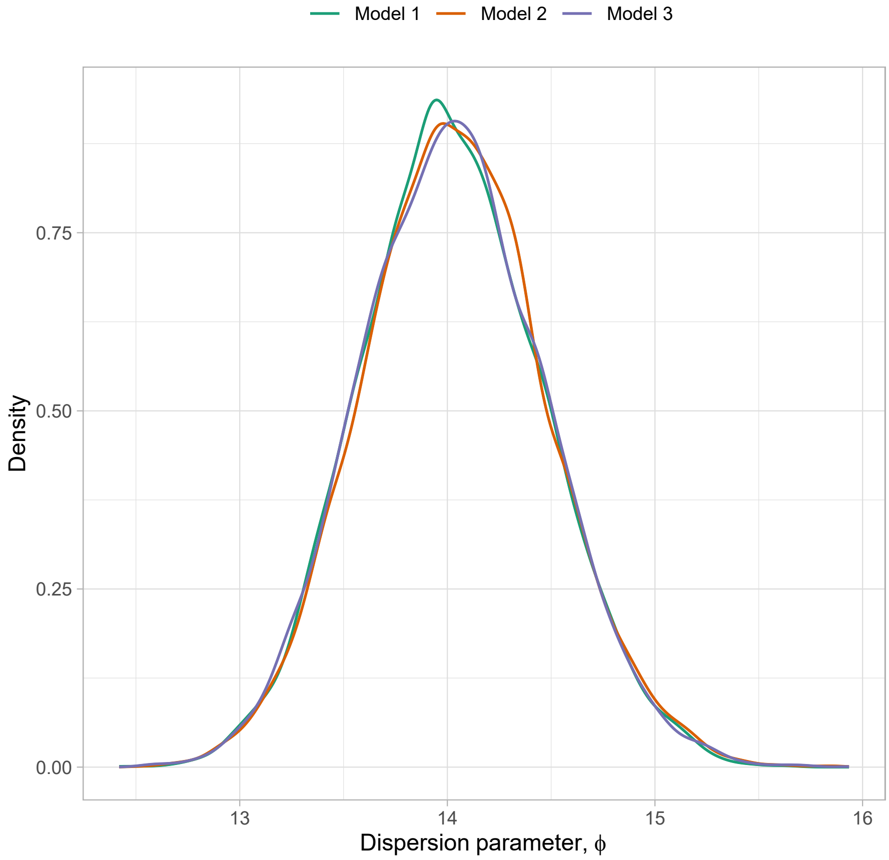

Figure S7: Inference for ϕ parameter for the three models favored in LOO cross-validation and WAIC.

2 Results

2.1 Number of contacts

The model we used may be described in the following generative model fashion.

$$\text{effect}_{\text{ind}} \sim \mathcal{N}(0, \tau)$$

$$\text{contact} \sim \text{Poisson}(\lambda)$$

$$\log(\lambda) = \alpha + \beta_{\text{age}}\text{age} + \beta_{\text{hh}}\text{household size} + \beta_{\text{date}}\text{date} + \beta_{\text{sex}}\text{sex} + \text{effect}_{\text{ind}}$$

$\text{effect}_{\text{ind}}$ indicates individual random effect used to account for the potential correlation among repeated measures for an individual.

Table S2: Estimates for the 2023 data that represent a typical week.

**Category**	**Covariates**	**No. Part**	**Mar mean (sd)**	**CR**	**Est. (95% CI)**
Age	0-4	60	5.31 (2.79)	1.00	1.00
	5-9	84	9.11 (4.72)	1.71	1.65 (1.37 - 1.99)
	10-14	96	9.47 (4.33)	1.78	1.71 (1.42 - 2.05)
	15-19	83	8.86 (4.06)	1.67	1.59 (1.31 - 1.91)
	20-29	256	3.32 (2.20)	0.63	0.60 (0.50 - 0.70)
	30-39	292	3.67 (2.11)	0.69	0.72 (0.61 - 0.85)
	40-49	352	4.22 (2.08)	0.79	0.80 (0.68 - 0.94)
	50-59	316	4.43 (2.49)	0.83	0.82 (0.70 - 0.97)
	60-69	273	5.28 (3.79)	0.99	1.00 (0.85 - 1.19)
	70+	166	5.25 (4.15)	0.99	1.05 (0.87 - 1.25)
Sex	Female	1036	4.94 (3.38)	1.00	1.00
	Male	951	5.01 (3.64)	1.01	0.97 (0.92 - 1.02)
Day	Sun	1987	3.45 (2.95)	1.00	1.00
	Mon	1987	5.21 (4.46)	1.51	1.51 (1.46 - 1.56)
	Tue	1987	5.15 (4.33)	1.49	1.49 (1.44 - 1.54)
	Wed	1987	6.09 (4.74)	1.77	1.76 (1.71 - 1.82)
	Thu	1987	5.53 (4.66)	1.60	1.61 (1.56 - 1.67)
	Fri	1987	5.47 (4.75)	1.59	1.59 (1.54 - 1.64)
	Sat	1987	3.90 (3.22)	1.13	1.13 (1.09 - 1.17)
HH size	1	254	3.52 (2.78)	1.00	1.00
	2	453	4.50 (3.26)	1.28	1.21 (1.10 - 1.32)
	3	583	5.06 (3.13)	1.44	1.33 (1.22 - 1.46)
	4	561	5.53 (3.72)	1.57	1.53 (1.40 - 1.67)
	5+	136	6.57 (4.77)	1.87	1.67 (1.49 - 1.89)

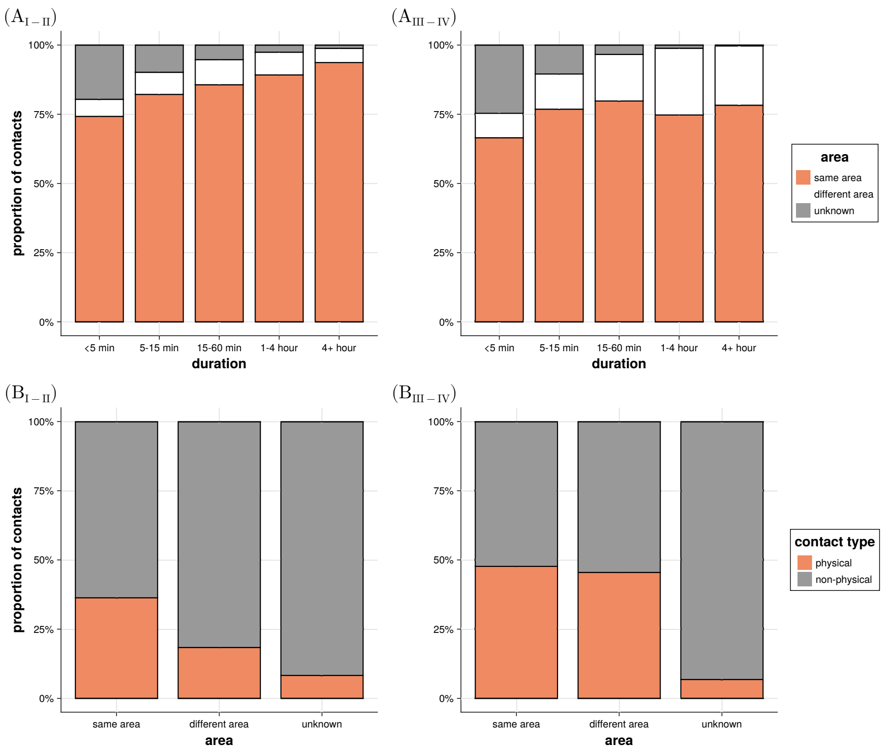

Figure S8: The proportion of social contacts which shows the correlation between (A) duration and residential area and (B) residential area and contact type. Subfigures (I-II) and (III-IV) show the proportion of contacts in Periods I-II, and III-IV, respectively.

Fig. S8($A_{\text{I-II}}$) and S8($A_{\text{III-IV}}$) show the correlation between the duration of close contacts and the residential area. In Periods I and II, the proportion of contacts with people in the same residential area increases with increasing duration. However, in Periods III and IV, despite an increase in the duration, the proportion of people in the same residential area remains consistent. We illustrated the correlation between the residential area and contact type in Fig. S8($B_{\text{I-II}}$) and S8($B_{\text{III-IV}}$). During Periods I and II, the proportion of non-physical contact with people in different residential areas is more significant than that in the same residential area. However, in Periods III and IV, the proportion of non-physical contacts remains consistent regardless of the residential area.

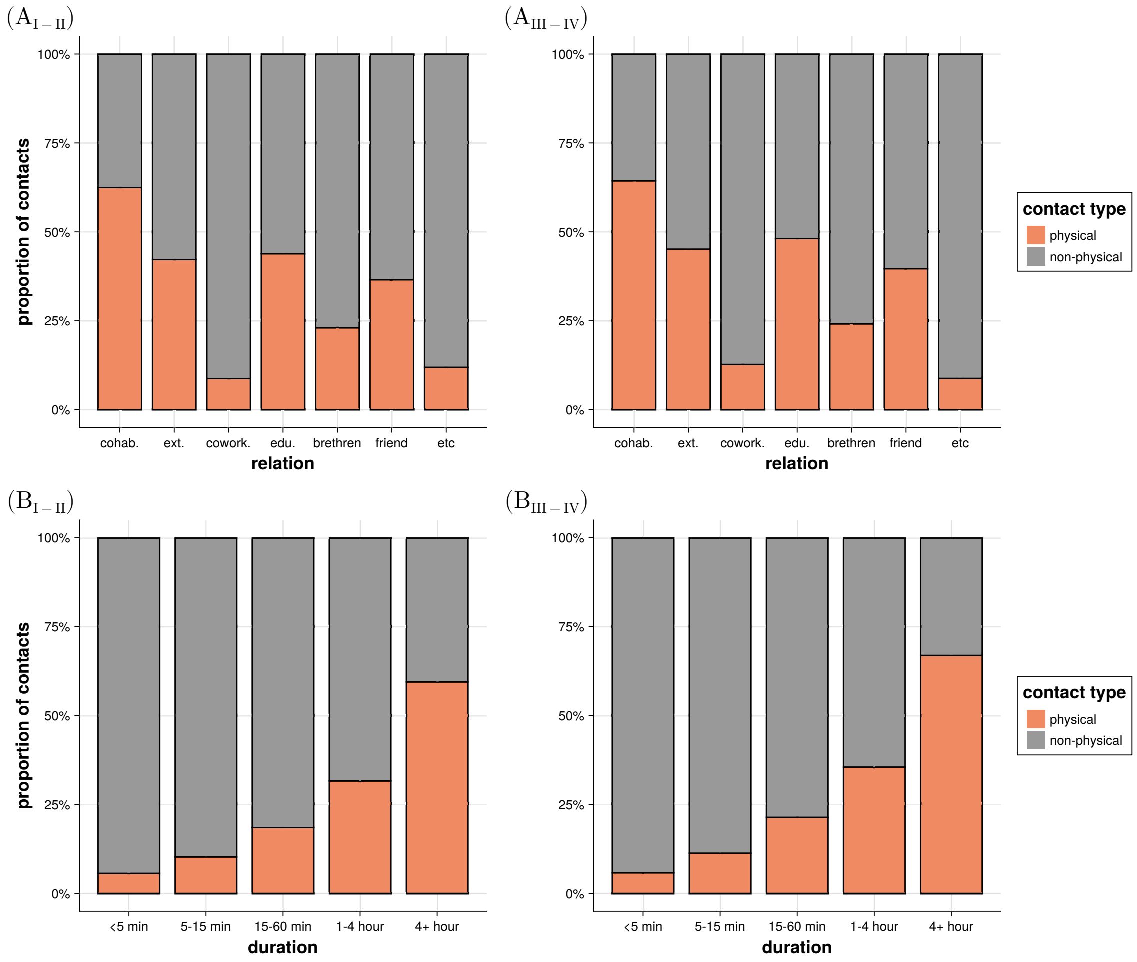

Figure S9: The proportion of social contacts which shows the correlation between (A) relation and contact type and (B) duration and contact type. Each (I-II) and (III-IV) shows the proportion of contacts in Periods I-II, and III-IV, respectively.

Fig. S9(A) and S9(B) explore the correlation between the contact type and relation, duration. Unlike the results of Fig. 2 and Fig. S8, where all correlations reveal different patterns over the period, the patterns of Fig. S9 are consistent across all periods. As shown in Fig. S9(A), physical contact is prevalent in interactions with cohabiting members and external family, educational relationships, and friends. In contrast, non-physical contacts dominate in interactions with coworkers, brethren, and etc relationships. Also, regardless of the period, as the duration of contact increases, the proportion of physical contacts increases, confirming the findings in Fig. 1(A) of Ref. [3].

The number of close contacts varies by residential area across different studies. Studies in Kenya [4] and Zimbabwe [5] show contrasting patterns of contact rates between rural and urban areas. This highlights geographical variations in contact patterns. Our results show fewer close contacts in metropolitan regions than other areas (see Table S8). A study in Southwest Uganda demonstrated increased contacts with travel distance from villages [6]. However, previous studies did not consider both participants' and contacts' residential areas. In this study, we recorded these across 17 administrative divisions. Contacts within the same area dominate, but during the Lunar New Year holidays, contacts between different regions increase due to family gatherings, enhancing inter-regional social mixing (see Table S6). These regional contact patterns, combined with mobility data, can improve infectious disease modeling.

Table S3: The number of contacts per day by period and relation

Period	Relation	Contacts per day	Percentage
Weekdays during the semester (I)	cohabiting member	2765.6	100
	extended family	358.8	100
	coworker	2697.4	100
	educational relationship	2147.0	100
	brethren	115.0	100
	friend	953.6	100
	etc	1873.6	100
Weekdays during the vacation (II)	cohabiting member	2859.6	103
	extended family	546.3	152
	coworker	2322.0	86
	educational relationship	1043.0	49
	brethren	109.3	95
	friend	1002.0	105
	etc	1848.0	99
Weekends (III)	cohabiting member	2982.5	108
	extended family	1080.0	301
	coworker	401.0	15
	educational relationship	290.5	14
	brethren	412.0	358
	friend	918.5	96
	etc	1213.0	65
Lunar New Year holidays (IV)	cohabiting member	3040.5	110
	extended family	4054.2	1130
	coworker	233.2	9
	educational relationship	93.2	4
	brethren	163.7	142
	friend	499.2	52
	etc	774.5	41

Table S4: The number of contacts per day by period and frequency

Period	Frequency	Contacts per day	Ratio (%)
Weekdays during the semester (I)	daily	2877.2	100
	5-6 per week	3468.0	100
	3-4 per week	1472.0	100
	1-2 per week	1143.4	100
	1-2 per month	667.8	100
	<1 per month	1282.6	100
Weekdays during the vacation (II)	daily	2877.6	100
	5-6 per week	2366.3	68
	3-4 per week	1483.0	101
	1-2 per week	1091.3	95
	1-2 per month	659.0	99
	<1 per month	1253.0	98
Weekends (III)	daily	2824.5	98
	5-6 per week	478.5	14
	3-4 per week	509.5	35
	1-2 per week	1088.5	95
	1-2 per month	940.5	141
	<1 per month	1456.0	114
Lunar New Year holidays (IV)	daily	2849.7	99
	5-6 per week	337.5	10
	3-4 per week	418.5	28
	1-2 per week	700.2	61
	1-2 per month	1175.5	176
	<1 per month	3377.2	263

Table S5: The number of contacts per day by period and duration

Period	Duration	Contacts per day	Ratio (%)
Weekdays during the semester (I)	<5 min	1017.6	100
	5-15 min	1335.4	100
	15-60 min	2115.8	100
	1-4 hour	2770.6	100
	4+ hour	3671.6	100
Weekdays during the vacation (II)	<5 min	982.0	97
	5-15 min	1169.0	88
	15-60 min	1763.0	83
	1-4 hour	2364.3	85
	4+ hour	3452.0	94
Weekends (III)	<5 min	633.5	62
	5-15 min	584.5	44
	15-60 min	1010.5	48
	1-4 hour	2004.0	72
	4+ hour	3065.0	83
Lunar New Year holidays (IV)	<5 min	488.0	48
	5-15 min	515.0	39
	15-60 min	970.5	46
	1-4 hour	2209.2	80
	4+ hour	4676.0	127

Table S6: The number of contacts per day by period and residential area

Period	Residential area	Contacts per day	Ratio (%)
Weekdays during the semester (I)	same	9600.6	100
	different	736.0	100
	unknown	574.4	100
Weekdays during the vacation (II)	same	8502.0	89
	different	752.0	102
	unknown	476.33	83
Weekends (III)	same	6294.0	66
	different	654.0	89
	unknown	349.5	61
Lunar New Year holidays (IV)	same	6446.0	67
	different	2202.5	299
	unknown	210.25	37

Table S7: The number of contacts per day by period and contact type

Period	Type	Contacts per day	Ratio (%)
Weekdays during the semester (I)	physical	3550.8	100
	non-physical	7360.2	100
Weekdays during the vacation (II)	physical	3463.3	98
	non-physical	6267.0	85
Weekends (III)	physical	3167.5	89
	non-physical	4130.0	56
Lunar New Year holidays (IV)	physical	4167.5	117
	non-physical	4691.2	64

Table S8: The contact rate for residential areas analyzed by grouping the areas into five major regions in South Korea. The metropolitan region includes Seoul, Incheon, and Gyeonggi.

Region	Contact rate
Metropolitan	4.84
Chungcheong	5.52
Gangwon	4.71
Youngnam	5.94
Honam	5.78

2.2 Age-grouped contact matrix

We calculated the age-grouped contact matrices. Each contact matrix was calculated using the bootstrap and considering reciprocity described in Method 2.2 (main paper). We bootstrapped 2000 iterations. In the contact matrix, each element represents the estimated average number of contacts per participant per day between age groups. The x and y-axis indicate the age group of the participants and contact people, respectively. The color bar means the daily contact number.

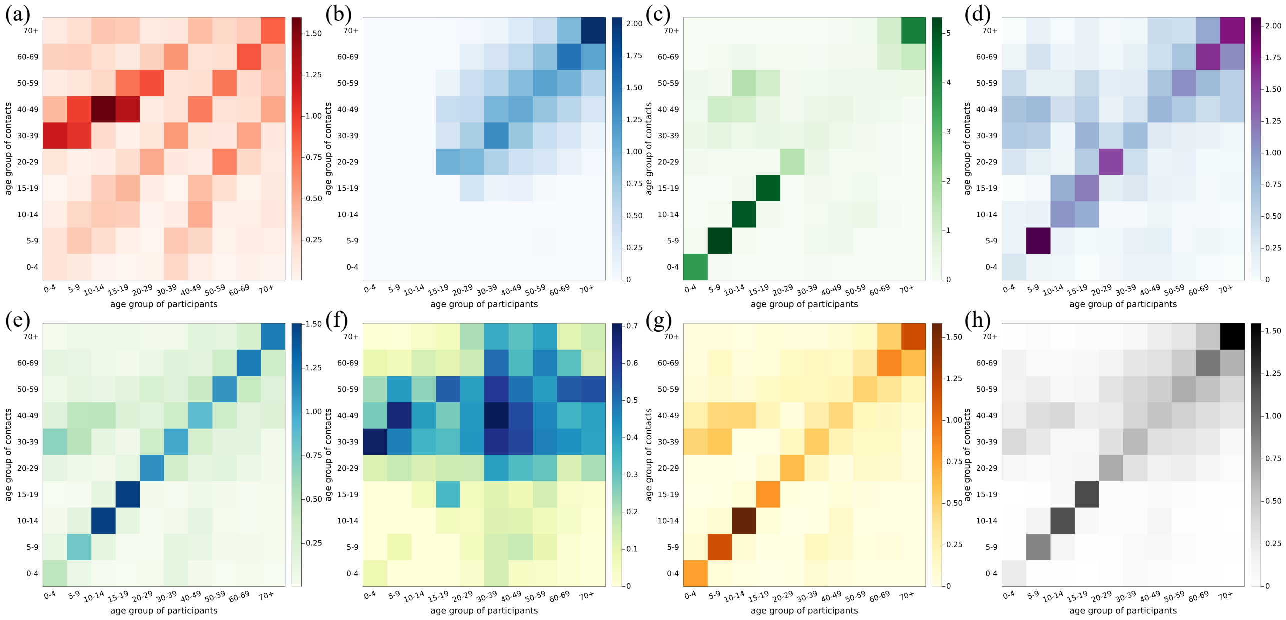

Figure S10: Contact matrix in each location. The contact matrices are the average daily contact number (a) at home, (b) in the workplace, (c) in the educational facility, (d) in the religious facility, (e) in the restaurant, (f) in the hospital, (g) in the outdoor, and (h) in the indoor. Each color bar has a different range.

Fig. S10 shows the contact matrices for each location where the social contacts occurred. The contact pattern at home is the most intergenerational. Contact is predominantly between groups of adults in the workplace and between groups of children and adolescents in the educational facility. In the hospitals, there is mixed intergenerational contact. The contact matrices in other locations are characterized by a highlighted diagonal component.

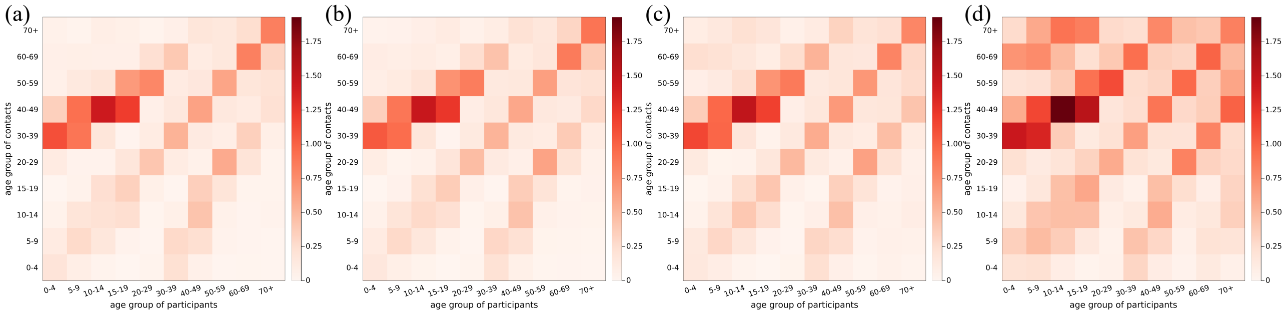

Figure S11: Contact matrix at home for each period. The contact matrices are the average daily contact number: (a) weekdays during the semester (Period I), (b) weekdays during the vacation (Period 2), (c) weekends (Period 3), and (d) Lunar New Year holidays (Period 4).

Fig. S11 shows that the number of contacts at home is higher on weekends and holidays (Periods III and IV) than on weekdays (Periods I and II). The secondary diagonal starting from the age group 30-39 is more prominent than the central diagonal. Fig. S11(d) unveils a third diagonal starting from the age group 60-69 and is reflected in the characteristics of the Lunar New Year holidays (Period 4).

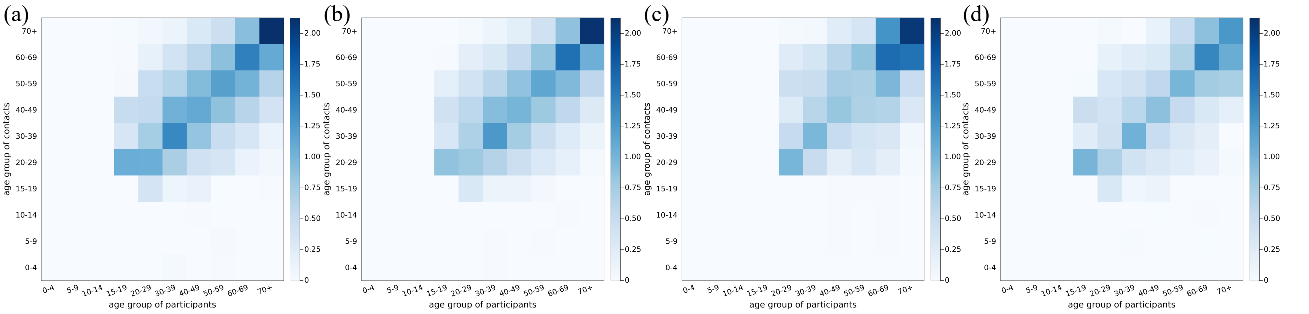

Figure S12: Contact matrix in the workplace for each period. The contact matrices are the average daily contact number: (a) weekdays during the semester (Period I), (b) weekdays during the vacation (Period 2), (c) weekends (Period 3), and (d) Lunar New Year holidays (Period 4).

Fig. S12 shows that the number of contacts in the workplace is higher on weekdays (Periods I and II) than on weekends and holidays (Periods III and IV), in contrast to the contact pattern at home.

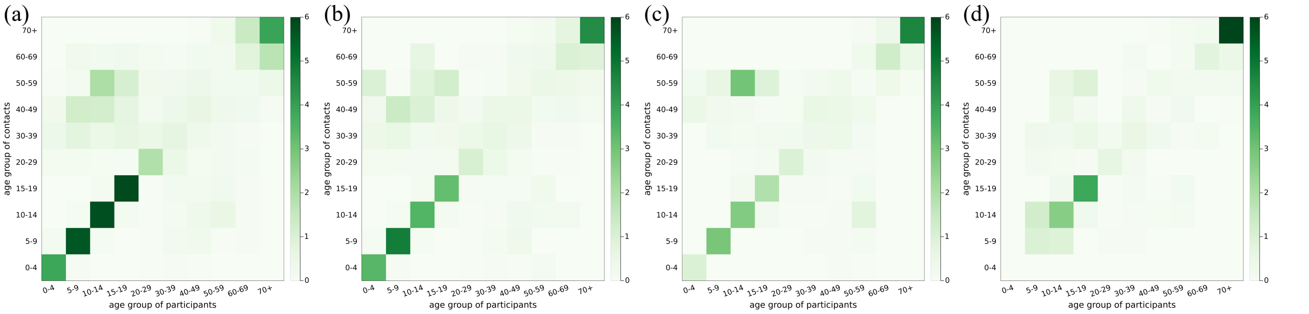

Figure S13: Contact matrix in the education facility for each period. The contact matrices are the average daily contact number: (a) weekdays during the semester (Period I), (b) weekdays during the vacation (Period 2), (c) weekends (Period 3), and (d) Lunar New Year holidays (Period 4).

Fig. S13 shows that the number of contacts in the educational facility is higher on weekdays (Period I) than on weekends and holidays (Periods III and IV). In this study, the educational facility includes schools and other facilities, such as swimming classes. So, there are also contacts between adult groups who are not minor groups. Contacts also occur on weekends and holidays in the educational facility for the same reason.

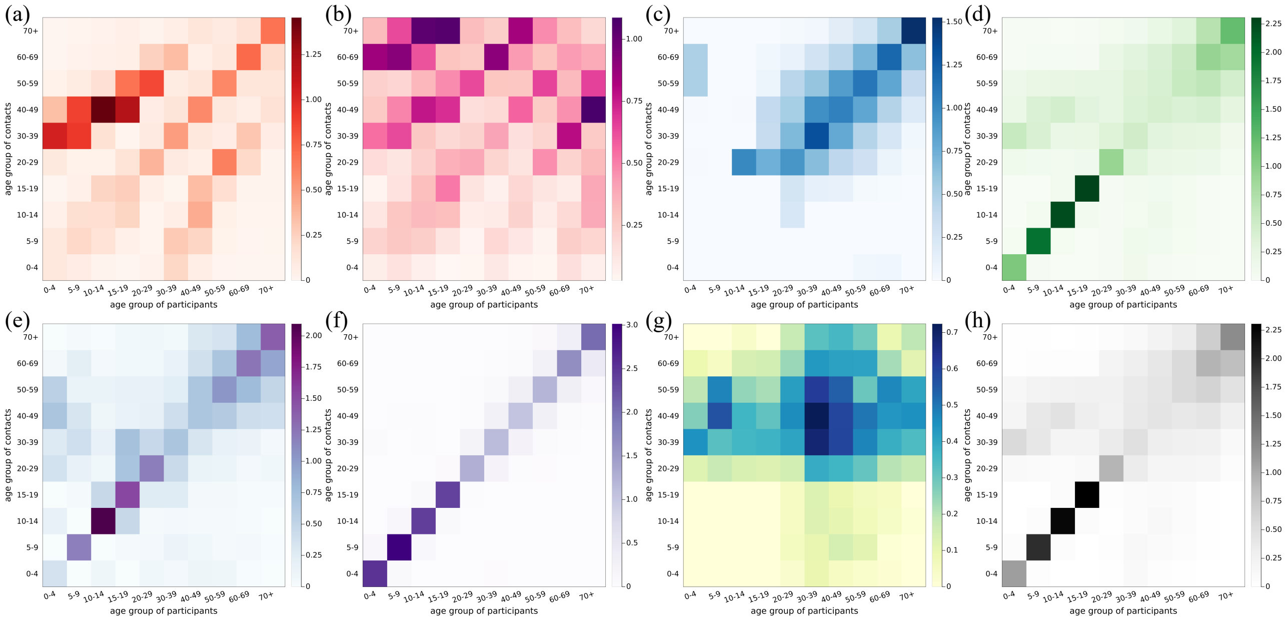

Figure S14: Contact matrix with each social relationship. The contact matrices are the average daily contact number by (a) the cohabiting member, (b) the extended family, (c) the coworker, (d) the educational relationship, (e) the brethren, (f) the friend, (g) the hospital relationship, and (h) the other. Each color bar has a different range.

Our survey did not allow multiple selections of social relationships with each contact. Although the relationships between people in society are multifaceted, our results show a limited contact matrix. Relationships and locations are closely related. The contact pattern of a social relationship associated with a particular location is very similar to the contact pattern of that location. For example, the contact pattern in the hospital facility is similar to that with hospital relationships (see Fig. S14(f) and S14(g)).

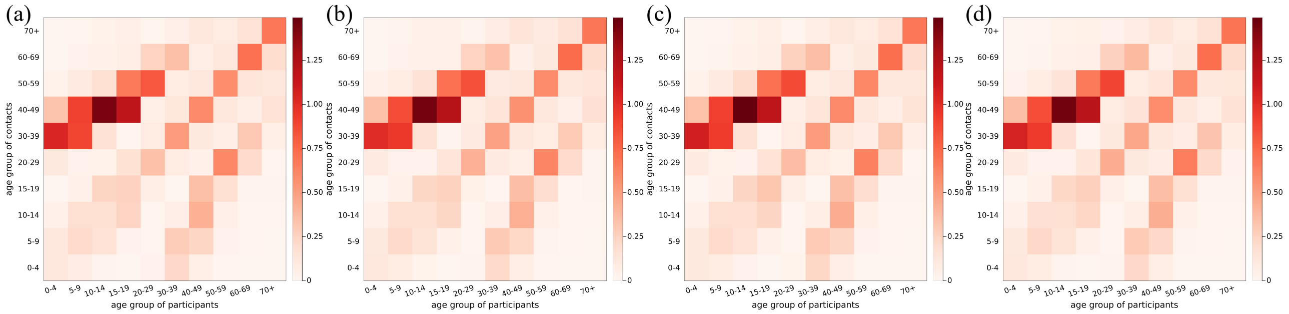

Figure S15: Contact matrix with the cohabiting member for each period. The contact matrices are the average daily contact number: (a) weekdays during the semester (Period I), (b) weekdays during the vacation (Period 2), (c) weekends (Period 3), and (d) Lunar New Year holidays (Period 4).

Fig. S15 shows the contact matrix with the cohabiting member. Regardless of the dates, all four Periods have the same contact patterns. Because the total survey period is short, it is unlikely that the household members will change during this period. Here, the cohabiting member is an individual living in the same house regardless of blood relationship.

Fig. S16 is the contact matrix with the extended family, leading to a higher contact number between older and younger people, such as grandparents and grandchildren. We see exceptionally high contact numbers during weekends and holidays, especially the Lunar New Year holidays (Period 4).

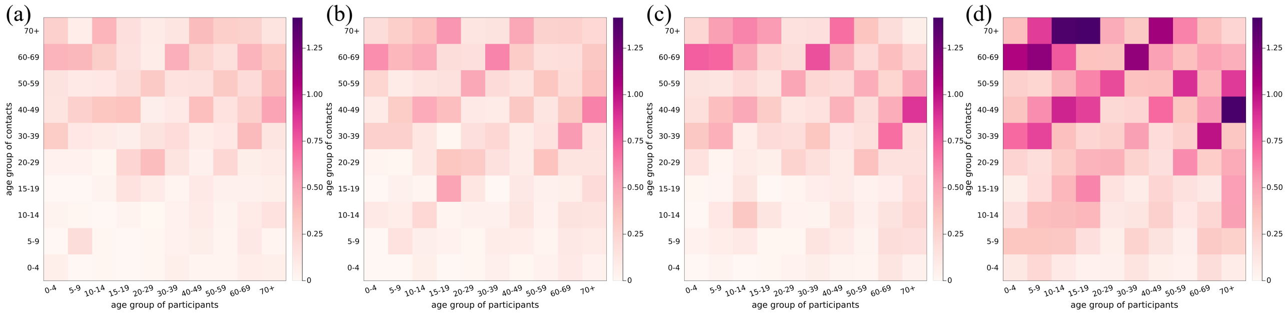

Figure S16: Contact matrix with the extended family for each period. The contact matrices are the average daily contact number: (a) weekdays during the semester (Period I), (b) weekdays during the vacation (Period 2), (c) weekends (Period 3), and (d) Lunar New Year holidays (Period 4).

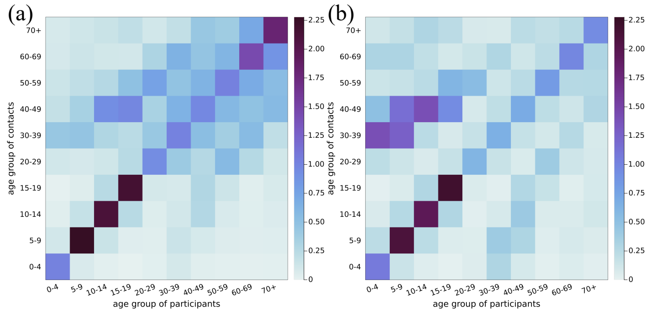

Figure S17: Contact matrix by the contact types. The contact matrices are the average daily contact number of the (a) non-physical and (b) physical contacts.

The survey included two types of contact: non-physical and physical (see Fig. S17). Contacts between minor groups tend to occur in the same pattern for both types of contact. Contacts between adult groups are more likely to be non-physical than physical. However, intergenerational contacts are more likely to be physical than non-physical.

Table S9: The age-grouped contact matrix value for Period I (Fig. 3(a) in main paper). Each value is the average of 2000 bootstraps. The values in parentheses are the 90% confidence interval.

**age group**	0-4	5-9	10-14	15-19	20-29	30-39	40-49	50-59	60-69	70+
0-4	2.2868<br>(0.0, 7.79)	0.1518<br>(0.0, 1.109)	0.0251<br>(0.0, 0.1)	0.0012<br>(0.0, 0.0)	0.0436<br>(0.0, 0.16)	0.2793<br>(0.01, 0.917)	0.0871<br>(0.0, 0.302)	0.0234<br>(0.0, 0.08)	0.0137<br>(0.0, 0.079)	0.0129<br>(0.0, 0.034)
5-9	0.2259<br>(0.0, 1.651)	5.7667<br>(0.672, 15.793)	0.2946<br>(0.0, 1.313)	0.0383<br>(0.0, 0.375)	0.0639<br>(0.0, 0.191)	0.4095<br>(0.0, 1.199)	0.3112<br>(0.0, 1.071)	0.0736<br>(0.0, 0.243)	0.0389<br>(0.0, 0.25)	0.0133<br>(0.0, 0.037)
10-14	0.0427<br>(0.0, 0.17)	0.3362<br>(0.0, 1.498)	6.0048<br>(1.06, 15.513)	0.4167<br>(0.0, 1.737)	0.0451<br>(0.0, 0.238)	0.1851<br>(0.0, 0.549)	0.5611<br>(0.093, 1.54)	0.1157<br>(0.0, 0.748)	0.0316<br>(0.0, 0.165)	0.0296<br>(0.0, 0.138)
15-19	0.002<br>(0.0, 0.0)	0.0434<br>(0.0, 0.425)	0.414<br>(0.0, 1.725)	5.9271<br>(0.134, 14.897)	0.1039<br>(0.0, 0.769)	0.117<br>(0.0, 0.412)	0.4494<br>(0.0, 1.422)	0.2304<br>(0.0, 1.063)	0.0348<br>(0.0, 0.164)	0.0263<br>(0.0, 0.047)
20-29	0.1989<br>(0.0, 0.728)	0.1957<br>(0.0, 0.584)	0.121<br>(0.0, 0.64)	0.2806<br>(0.0, 2.076)	1.2591<br>(0.0, 4.433)	0.5108<br>(0.0, 2.345)	0.302<br>(0.0, 1.691)	0.6346<br>(0.0, 2.091)	0.2566<br>(0.0, 1.536)	0.0759<br>(0.0, 0.501)
30-39	1.3294<br>(0.049, 4.363)	1.3093<br>(0.001, 3.832)	0.5184<br>(0.0, 1.538)	0.3297<br>(0.0, 1.16)	0.5331<br>(0.0, 2.447)	1.4341<br>(0.0, 4.305)	0.718<br>(0.0, 2.737)	0.4346<br>(0.0, 1.796)	0.5197<br>(0.0, 2.236)	0.1666<br>(0.0, 0.942)
40-49	0.5021<br>(0.0, 1.74)	1.2053<br>(0.0, 4.148)	1.9037<br>(0.316, 5.226)	1.5347<br>(0.0, 4.857)	0.3819<br>(0.0, 2.138)	0.8698<br>(0.0, 3.316)	1.4014<br>(0.0, 4.003)	0.7948<br>(0.0, 2.895)	0.5375<br>(0.0, 2.547)	0.4616<br>(0.0, 2.212)
50-59	0.1451<br>(0.0, 0.499)	0.3072<br>(0.0, 1.016)	0.4231<br>(0.0, 2.735)	0.8477<br>(0.0, 3.911)	0.8645<br>(0.0, 2.849)	0.5672<br>(0.0, 2.344)	0.8563<br>(0.0, 3.119)	1.4972<br>(0.0, 4.243)	0.902<br>(0.0, 3.482)	0.5691<br>(0.0, 2.624)
60-69	0.0747<br>(0.0, 0.43)	0.1424<br>(0.0, 0.913)	0.1014<br>(0.0, 0.527)	0.1122<br>(0.0, 0.53)	0.3065<br>(0.0, 1.835)	0.5949<br>(0.0, 2.559)	0.5078<br>(0.0, 2.407)	0.791<br>(0.0, 3.054)	2.046<br>(0.0, 5.782)	1.0034<br>(0.0, 4.122)
70+	0.058<br>(0.0, 0.151)	0.0402<br>(0.0, 0.111)	0.0784<br>(0.0, 0.364)	0.0699<br>(0.0, 0.126)	0.0748<br>(0.0, 0.494)	0.1573<br>(0.0, 0.89)	0.3598<br>(0.0, 1.724)	0.4117<br>(0.0, 1.898)	0.8277<br>(0.0, 3.4)	2.3766<br>(0.0, 6.847)

Table S10: The age-grouped contact matrix value for Period 3 (Fig. 3(b) in main paper). Each value is the average of 2000 bootstraps. The values in parentheses are the 90% confidence interval.

**age group**	0-4	5-9	10-14	15-19	20-29	30-39	40-49	50-59	60-69	70+
0-4	1.9708 (0.0, 7.115)	0.1422 (0.0, 1.015)	0.0305 (0.0, 0.199)	0.0 (0.0, 0.0)	0.0461 (0.0, 0.177)	0.276 (0.016, 0.917)	0.0828 (0.0, 0.285)	0.0311 (0.0, 0.086)	0.0214 (0.0, 0.109)	0.0074 (0.0, 0.003)
5-9	0.2117 (0.0, 1.51)	4.508 (0.001, 14.095)	0.2731 (0.0, 1.41)	0.0464 (0.0, 0.385)	0.0542 (0.0, 0.19)	0.4222 (0.0, 1.32)	0.2824 (0.0, 1.01)	0.0597 (0.0, 0.237)	0.0273 (0.0, 0.115)	0.0149 (0.0, 0.014)
10-14	0.0519 (0.0, 0.337)	0.3118 (0.0, 1.61)	3.413 (0.0, 11.163)	0.3741 (0.0, 1.838)	0.0493 (0.0, 0.261)	0.1186 (0.0, 0.373)	0.4887 (0.035, 1.344)	0.0862 (0.0, 0.654)	0.0427 (0.0, 0.202)	0.0265 (0.0, 0.126)
15-19	0.0 (0.0, 0.0)	0.0526 (0.0, 0.437)	0.3717 (0.0, 1.826)	3.158 (0.0, 10.244)	0.1029 (0.0, 0.636)	0.0907 (0.0, 0.32)	0.3988 (0.0, 1.16)	0.1946 (0.0, 0.886)	0.0303 (0.0, 0.13)	0.0449 (0.0, 0.219)
20-29	0.2104 (0.0, 0.805)	0.1661 (0.0, 0.582)	0.1323 (0.0, 0.701)	0.2781 (0.0, 1.717)	0.8953 (0.0, 3.009)	0.4284 (0.0, 1.996)	0.2856 (0.0, 1.504)	0.6197 (0.0, 1.861)	0.2711 (0.0, 1.649)	0.078 (0.0, 0.524)
30-39	1.3137 (0.077, 4.363)	1.3498 (0.0, 4.221)	0.3321 (0.0, 1.044)	0.2558 (0.0, 0.903)	0.4471 (0.0, 2.083)	1.254 (0.0, 4.02)	0.6105 (0.0, 2.237)	0.4195 (0.0, 1.785)	0.5204 (0.0, 2.216)	0.2089 (0.0, 1.475)
40-49	0.4776 (0.0, 1.645)	1.0938 (0.0, 3.913)	1.658 (0.119, 4.559)	1.3617 (0.0, 3.96)	0.3611 (0.0, 1.901)	0.7395 (0.0, 2.71)	1.235 (0.0, 3.405)	0.6894 (0.0, 2.629)	0.5181 (0.0, 2.45)	0.4611 (0.0, 2.57)
50-59	0.193 (0.0, 0.534)	0.249 (0.0, 0.988)	0.3152 (0.0, 2.392)	0.7162 (0.0, 3.26)	0.8441 (0.0, 2.535)	0.5475 (0.0, 2.33)	0.7427 (0.0, 2.832)	1.4102 (0.0, 3.88)	0.8749 (0.0, 3.167)	0.625 (0.0, 2.607)
60-69	0.1164 (0.0, 0.596)	0.0998 (0.0, 0.421)	0.137 (0.0, 0.648)	0.0978 (0.0, 0.419)	0.3239 (0.0, 1.97)	0.5956 (0.0, 2.537)	0.4895 (0.0, 2.315)	0.7672 (0.0, 2.777)	2.0372 (0.0, 5.711)	1.0306 (0.0, 3.901)
70+	0.0335 (0.0, 0.013)	0.045 (0.0, 0.043)	0.07 (0.0, 0.332)	0.1196 (0.0, 0.583)	0.0769 (0.0, 0.516)	0.1972 (0.0, 1.393)	0.3594 (0.0, 2.003)	0.4521 (0.0, 1.886)	0.8502 (0.0, 3.218)	2.5727 (0.0, 7.671)

Table S11: The age-grouped contact matrix value for Period 2 (Fig. 3(c) in main paper). Each value is the average of 2000 bootstraps. The values in parentheses are the 90% confidence interval.

**age group**	0-4	5-9	10-14	15-19	20-29	30-39	40-49	50-59	60-69	70+
0-4	0.2863<br>(0.0, 1.855)	0.1172<br>(0.0, 0.922)	0.0276<br>(0.0, 0.112)	0.0025<br>(0.0, 0.0)	0.0279<br>(0.0, 0.109)	0.2471<br>(0.002, 0.872)	0.0748<br>(0.0, 0.37)	0.016<br>(0.0, 0.08)	0.0465<br>(0.0, 0.182)	0.0197<br>(0.0, 0.101)
5-9	0.1745<br>(0.0, 1.372)	0.8497<br>(0.0, 3.957)	0.2377<br>(0.0, 1.171)	0.0484<br>(0.0, 0.394)	0.0231<br>(0.0, 0.101)	0.3353<br>(0.0, 1.247)	0.2785<br>(0.0, 1.088)	0.0457<br>(0.0, 0.213)	0.0703<br>(0.0, 0.589)	0.0582<br>(0.0, 0.386)
10-14	0.0469<br>(0.0, 0.19)	0.2713<br>(0.0, 1.337)	1.5904<br>(0.0, 5.224)	0.3459<br>(0.0, 1.543)	0.028<br>(0.0, 0.201)	0.0949<br>(0.0, 0.416)	0.4825<br>(0.025, 1.373)	0.0814<br>(0.0, 0.635)	0.0559<br>(0.0, 0.561)	0.0816<br>(0.0, 0.746)
15-19	0.0042<br>(0.0, 0.0)	0.0549<br>(0.0, 0.447)	0.3436<br>(0.0, 1.533)	1.5435<br>(0.0, 5.278)	0.0828<br>(0.0, 0.618)	0.0426<br>(0.0, 0.242)	0.3596<br>(0.0, 1.089)	0.1915<br>(0.0, 0.95)	0.0343<br>(0.0, 0.161)	0.0703<br>(0.0, 0.652)
20-29	0.1271<br>(0.0, 0.498)	0.0706<br>(0.0, 0.311)	0.0751<br>(0.0, 0.539)	0.2236<br>(0.0, 1.669)	0.897<br>(0.0, 3.404)	0.2681<br>(0.0, 1.226)	0.1303<br>(0.0, 0.896)	0.5957<br>(0.0, 1.752)	0.2288<br>(0.0, 1.381)	0.0896<br>(0.0, 0.592)
30-39	1.176<br>(0.008, 4.151)	1.0719<br>(0.0, 3.987)	0.2658<br>(0.0, 1.166)	0.12<br>(0.0, 0.681)	0.2798<br>(0.0, 1.28)	0.9189<br>(0.0, 3.22)	0.272<br>(0.0, 1.202)	0.2254<br>(0.0, 1.068)	0.509<br>(0.0, 2.242)	0.1549<br>(0.0, 1.111)
40-49	0.4314<br>(0.0, 2.134)	1.0786<br>(0.0, 4.215)	1.637<br>(0.086, 4.66)	1.228<br>(0.0, 3.721)	0.1647<br>(0.0, 1.133)	0.3295<br>(0.0, 1.456)	1.0214<br>(0.0, 3.029)	0.4<br>(0.0, 1.956)	0.3634<br>(0.0, 1.645)	0.5392<br>(0.0, 2.535)
50-59	0.0995<br>(0.0, 0.495)	0.1906<br>(0.0, 0.887)	0.2975<br>(0.0, 2.323)	0.7046<br>(0.0, 3.496)	0.8115<br>(0.0, 2.386)	0.2942<br>(0.0, 1.393)	0.431<br>(0.0, 2.107)	1.2988<br>(0.0, 4.366)	0.5881<br>(0.0, 2.652)	0.4582<br>(0.0, 2.213)
60-69	0.2533<br>(0.0, 0.994)	0.2571<br>(0.0, 2.155)	0.1792<br>(0.0, 1.798)	0.1108<br>(0.0, 0.52)	0.2733<br>(0.0, 1.649)	0.5825<br>(0.0, 2.566)	0.3434<br>(0.0, 1.555)	0.5157<br>(0.0, 2.326)	1.7067<br>(0.0, 5.582)	0.8259<br>(0.0, 3.492)
70+	0.0887<br>(0.0, 0.453)	0.1756<br>(0.0, 1.164)	0.2157<br>(0.0, 1.972)	0.1871<br>(0.0, 1.734)	0.0883<br>(0.0, 0.583)	0.1462<br>(0.0, 1.049)	0.4203<br>(0.0, 1.976)	0.3315<br>(0.0, 1.601)	0.6813<br>(0.0, 2.88)	1.7724<br>(0.0, 5.089)

Table S12: The age-grouped contact matrix value for Period 4 (Fig. 3(d) in main paper). Each value is the average of 2000 bootstraps. The values in parentheses are the 90% confidence interval.

**age group**	0-4	5-9	10-14	15-19	20-29	30-39	40-49	50-59	60-69	70+
0-4	0.2345 (0.0, 1.201)	0.2373 (0.0, 1.195)	0.0944 (0.0, 0.659)	0.0271 (0.0, 0.08)	0.0561 (0.0, 0.217)	0.3041 (0.002, 1.026)	0.1017 (0.0, 0.478)	0.0359 (0.0, 0.155)	0.1326 (0.0, 0.714)	0.0582 (0.0, 0.222)
5-9	0.3533 (0.0, 1.779)	0.6119 (0.0, 2.385)	0.3865 (0.0, 1.882)	0.1469 (0.0, 1.067)	0.0479 (0.0, 0.173)	0.4284 (0.0, 1.573)	0.3064 (0.0, 1.102)	0.0638 (0.0, 0.245)	0.2125 (0.0, 0.972)	0.1904 (0.0, 0.936)
10-14	0.1605 (0.0, 1.12)	0.4412 (0.0, 2.148)	0.8806 (0.0, 3.51)	0.4886 (0.0, 2.216)	0.0806 (0.0, 0.377)	0.1414 (0.0, 0.863)	0.57 (0.062, 1.749)	0.1063 (0.0, 0.772)	0.1496 (0.0, 0.825)	0.3317 (0.0, 1.52)
15-19	0.0458 (0.0, 0.135)	0.1666 (0.0, 1.21)	0.4854 (0.0, 2.202)	1.294 (0.0, 4.66)	0.1352 (0.0, 0.925)	0.0707 (0.0, 0.345)	0.4595 (0.0, 1.514)	0.2497 (0.0, 1.199)	0.078 (0.0, 0.695)	0.3102 (0.0, 1.519)
20-29	0.2557 (0.0, 0.991)	0.1466 (0.0, 0.53)	0.2162 (0.0, 1.011)	0.3651 (0.0, 2.499)	0.7613 (0.0, 2.9)	0.2803 (0.0, 1.458)	0.1748 (0.0, 1.025)	0.7652 (0.0, 2.344)	0.3162 (0.0, 1.722)	0.2614 (0.0, 1.835)
30-39	1.4473 (0.009, 4.886)	1.3697 (0.0, 5.03)	0.396 (0.0, 2.416)	0.1994 (0.0, 0.972)	0.2925 (0.0, 1.522)	0.8216 (0.0, 2.672)	0.2656 (0.0, 1.414)	0.2662 (0.0, 1.397)	0.797 (0.0, 2.422)	0.2845 (0.0, 1.546)
40-49	0.5864 (0.0, 2.753)	1.1867 (0.0, 4.266)	1.934 (0.21, 5.934)	1.5691 (0.0, 5.169)	0.221 (0.0, 1.296)	0.3218 (0.0, 1.713)	1.0254 (0.0, 3.0)	0.4015 (0.0, 1.926)	0.4363 (0.0, 2.292)	1.0237 (0.0, 3.283)
50-59	0.2231 (0.0, 0.962)	0.2662 (0.0, 1.023)	0.3887 (0.0, 2.822)	0.9187 (0.0, 4.411)	1.0424 (0.0, 3.194)	0.3474 (0.0, 1.823)	0.4326 (0.0, 2.075)	1.239 (0.0, 3.845)	0.5373 (0.0, 2.552)	0.7326 (0.0, 3.006)
60-69	0.7226 (0.0, 3.889)	0.7775 (0.0, 3.558)	0.4795 (0.0, 2.646)	0.2517 (0.0, 2.244)	0.3778 (0.0, 2.058)	0.9122 (0.0, 2.772)	0.4122 (0.0, 2.166)	0.4712 (0.0, 2.238)	1.452 (0.0, 4.419)	0.7668 (0.0, 3.193)
70+	0.2614 (0.0, 0.998)	0.5747 (0.0, 2.826)	0.8772 (0.0, 4.021)	0.8256 (0.0, 4.042)	0.2576 (0.0, 1.809)	0.2686 (0.0, 1.46)	0.7979 (0.0, 2.559)	0.53 (0.0, 2.175)	0.6325 (0.0, 2.634)	1.4385 (0.0, 4.26)

2.3 Contact patterns compared to the pre-pandemic and pandemic level

To compare the level of contacts between the pre-pandemic and pandemic periods, we employed the next generation approach [7, 8, 9, 10, 11, 12]. Assuming uniform susceptibility and infectiousness across all age groups, the relative reduction in contact numbers was assessed by comparing the spectral radii (dominant eigenvalues) of the contact matrices for the two periods:

$$\tau = \frac{\rho\left(\left[c_{ij}^{2023} N_i^{2023}\right]_{1\le i,j\le n}\right)}{\rho\left(\left[c_{ij}^{pre} N_i^{pre}\right]_{1\le i,j\le n}\right)},$$

where ρ denotes the dominant eigenvalue of the respective contact rates. Here, c_{ij}^{pre} represents contact rates projected from pre-pandemic data [13], while c_{ij}^{2023} is derived from the our survey results. N_i denotes the population size specific to each age group.

Table S13: Ratio of the change in the maximum eigenvalues of next generation matrix from the contact numbers over the four periods, compared to data reflecting the previous findings [13].

**Period**	τ
I	0.6985
II	0.6650
III	0.5150
IV	0.5851

Table S14: Relative contact rates by age group in POLYMOD data, the 2020 survey, and the current data. The values in the "POLYMOD Data" and "2020 Survey Data" columns were extracted directly from Ref. [14].

**Age group**	**POLYMOD Data**†	**2020 Survey Data**	**Current Data**
0-4	1.00	1.00	1.00
5-9	1.42 (1.29-1.57)	0.98 (0.69-1.38)	1.43 (1.21-1.69)
10-14	1.75 (1.59-1.93)	1.29 (0.83-2.00)	1.38 (1.16-1.63)
15-19	1.69 (1.54-1.86)	1.28 (0.83-1.97)	1.28 (1.08-1.52)
20-29	1.46 (1.34-1.59)	1.15 (0.74-1.78)	0.52 (0.45-0.61)
30-39	1.46 (1.34-1.58)	1.09 (0.69-1.73)	0.62 (0.53-0.72)
40-49	1.39 (1.28-1.51)	1.07 (0.68-1.71)	0.70 (0.60-0.81)
50-59	1.32 (1.21-1.43)	1.18 (0.74-1.89)	0.75 (0.65-0.87)
60-69	1.32 (1.21-1.43)	1.12 (0.70-1.80)	0.91 (0.78-1.06)
70+	0.81 (0.73-0.88)	1.04 (0.64-1.69)	1.04 (0.88-1.23)

†projected

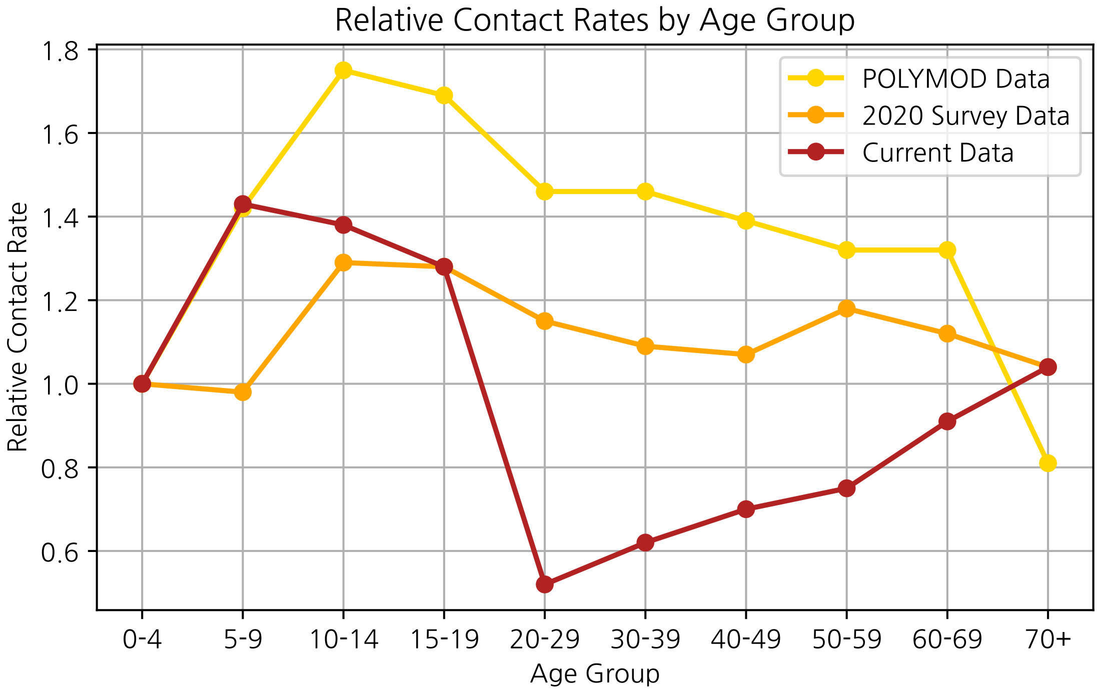

Figure S18: Relative contact rates by age group based on POLYMOD data (yellow), 2020 survey data (orange), and current data (red). The contact rates demonstrate the variations in social contact patterns over time, highlighting changes observed during the transition from pre-pandemic to current conditions. Note that the values are relative to the baseline age group 0-4.

References

[1] Paul-Christian Bürkner. brms: An r package for bayesian multilevel models using stan. *Journal of statistical software*, 80:1–28, 2017.

[2] Aki Vehtari, Jonah Gabry, and Andrew Gelman. Practical bayesian model evaluation using leave-one-out cross-validation and waic. *Statistics and Computing*, 27:1413–1432, 2017.

[3] Joël Mossong, Niel Hens, Mark Jit, Philippe Beutels, Kari Auranen, Rafael Mikolajczyk, Marco Massari, Stefania Salmaso, Gianpaolo Scalia Tomba, Jacco Wallinga, et al. Social contacts and mixing patterns relevant to the spread of infectious diseases. *PLoS medicine*, 5(3):e74, 2008.

[4] Moses Chapa Kiti, Timothy Muiruri Kinyanjui, Dorothy Chelagat Koech, Patrick Kiio Munywoki, Graham Francis Medley, and David James Nokes. Quantifying age-related rates of social contact using diaries in a rural coastal population of kenya. *PloS one*, 9(8):e104786, 2014.

[5] Alessia Melegaro, Emanuele Del Fava, Piero Poletti, Stefano Merler, Constance Nyamukapa, John Williams, Simon Gregson, and Piero Manfredi. Social contact structures and time use patterns in the manicaland province of zimbabwe. *PloS one*, 12(1):e0170459, 2017.

[6] O Le Polain de Waroux, Sandra Cohuet, Donny Ndazima, AJ Kucharski, Aitana Juan-Giner, Stefan Flasche, Elioda Tumwesigye, Rinah Arinaitwe, Juliet Mwanga-Amumpaire, Yap Boum, et al. Characteristics of human encounters and social mixing patterns relevant to infectious diseases spread by close contact: a survey in southwest uganda. *BMC infectious diseases*, 18:1–12, 2018.

[7] Odo Diekmann, Johan Andre Peter Heesterbeek, and Johan Anton Jacob Metz. On the definition and the computation of the basic reproduction ratio r 0 in models for infectious diseases in heterogeneous populations. *Journal of mathematical biology*, 28:365–382, 1990.

[8] Jacco Wallinga, Peter Teunis, and Mirjam Kretzschmar. Using data on social contacts to estimate age-specific transmission parameters for respiratory-spread infectious agents. *American Journal of Epidemiology*, 164(10):936–944, 2006.

[9] Petra Klepac, Adam J Kucharski, Andrew JK Conlan, Stephen Kissler, Maria L Tang, Hannah Fry, and Julia R Gog. Contacts in context: large-scale setting-specific social mixing matrices from the bbc pandemic project. *MedRxiv*, pages 2020–02, 2020.

[10] Juanjuan Zhang, Maria Litvinova, Yuxia Liang, Yan Wang, Wei Wang, Shanlu Zhao, Qianhui Wu, Stefano Merler, Cécile Viboud, Alessandro Vespignani, et al. Changes in contact patterns shape the dynamics of the covid-19 outbreak in china. *Science*, 368(6498):1481–1486, 2020.

[11] Christopher I Jarvis, Kevin Van Zandvoort, Amy Gimma, Kiesha Prem, Petra Klepac, G James Rubin, and W John Edmunds. Quantifying the impact of physical distance measures on the transmission of covid-19 in the uk. *BMC medicine*, 18:1–10, 2020.

[12] Jantien A Backer, Eric RA Vos, Gerco den Hartog, Cheyenne CE van Hagen, Hester E de Melker, Fiona RM van der Klis, and Jacco Wallinga. Contact behaviour before, during and after the covid-19 pandemic in the netherlands: evidence from contact surveys in 2016-2017 and 2020-2023. *medRxiv*, pages 2024–03, 2024.

[13] Kiesha Prem, Alex R Cook, and Mark Jit. Projecting social contact matrices in 152 countries using contact surveys and demographic data. *PLoS computational biology*, 13(9):e1005697, 2017.

[14] Jun Sup Yum. Monitoring of infectious disease contacts and development of early response system based on information communication technology, Health Technology R&D Project, 2020-01-31–2021-12-31, HG20C0003. https://library.nih.go.kr/ncmiklib/archive/rom/reportView.do, 2020.
