# Supplementary material for: Social contact patterns in South Korea: an analysis of a survey conducted in 2023-2024
Source: BMC Infect Dis. 2025 Mar 1;25:295. doi: 10.1186/s12879-025-10706-y (PMC11871801; doi:10.1186/s12879-025-10706-y)
Supplement: Supplementary file 1 — Supplementary Material 1. Details on statistical analysis, including survey design, contact numbers, age-grouped contact matrix, and comparison of contact patterns between pre-pandemic and pandemic levels. [file 12879_2025_10706_MOESM1_ESM.zip › survey_offline_v3_ENG.pdf]

Gallup

GALLUP INTERNATIONAL

A1-5

Survey of possible social contacts for respiratory infectious disease transmission (Offline ver.)

PJ# :

Hello, we are from the Healthcare Research Office at Gallup Korea. We are conducting a survey on behalf of the National Institute of Mathematical Sciences (NIMS) on the topic of social contacts for possible transmission of respiratory infectious diseases. The results from the survey is anonymous and will be handled following the Data Protection Act and the guidelines of the KOrean Society for Opinion & MArketing Research (KOSOMAR). We kindly ask for your cooperation.

Pre-survey

SQ 1) What year were you born?

____________ year ──▶ ____________-year-old

SQ 2) What is your sex?

1. Male   2. Female

SQ 3) Which region do you currently reside in?

1. Seoul	2. Busan	3. Deagu	4. Incheon	5. Gwangju	6. Deajeon
7. Ulsan	8. Sejong	9. Gyeonggi	10. Gangwon	11. Chungcheongbuk	12. Chungcheongnam
13. Jeollabuk	14. Jeollanam	15. Gyeongsangbuk	16. Gyeongsangnam	17. Jeju	

※ Survey description

This is a two-round survey. You will be asked to record information about everyone with whom you had social contact for seven days in each round. The first round will run from Wednesday, December 6, to Tuesday, December 12, 2023, and the second round will run from Wednesday, February 7, to Tuesday, February 13, 2024. Please read the following information slowly and carefully to confirm your willingness to participate in the survey.

※ How to take in the survey

For seven days, record information about everyone with whom you had social contact from 00:00 to 24:00 on a separate record-keeping form that you downloaded and will later enter into the survey link. Social contact is defined as an interactive dialogue consisting of three or more words or skin contact (e.g., a handshake, hug, kiss, or contact sports such as football or basketball). The required information includes age, sex, relationship, location, frequency, and duration, with specific details listed below.
Please read the following points slowly and carefully before responding to indicate your willingness to participate in the survey.

※ Tips for participating in the survey

[Definition of a day]
- ▶ 00:00 to 24:00 on the specified date
  Ex) If the date to respond is August 5, then record from 00:00 to 24:00 on 5 August.

[Criteria for social contact]
- ▶ Interactive dialogue consisting of three or more words or skin contact (i.e., a handshake, hug, kiss, or contact sports such as football or basketball)

[Notes]
- ▶ Do not fill in if contact was only by mobile or online

[Information required about the contact person you talked to or had contact with]
- ▶ Age of the contact person
- ▶ sex of the contact person
- ▶ Social relationship with you
- ▶ Location of contact
- ▶ Residence of the contact person
- ▶ Frequency of contact
- ▶ Duration of contact
- ▶ Size of contact

SQ 4) Do you have a clear understanding of how to participate in the survey described?

1. Yes   2. No ⇢ Recruitment closed   3. I'm not sure. ⇢ Recruitment closed

SQ 5) If yes, would you be willing to participate in the survey?

1. Yes (Participating)   2. No (Not Participating) ⇢ Recruitment closed

SQ 6) The survey will be conducted over two seven-day periods: Wednesday, December 6, to Tuesday, December 12, 2023, and the second round from Wednesday, February 7, to Tuesday, February 13, 2024. You should record your daily contacts each day on the form. After that, you either enter it online yourself, or submit it to an investigator. Are you eligible to participate in the survey?

1. Yes   2. No ⇢ Recruitment closed

Survey - Participant information

DQ 1) What is your occupation?

1. Self-employed (small business owner with nine or fewer employees, family worker, etc.)
2. Sale/service (shop clerk, salesperson, etc.)
3. Skilled/craft worker (driver, lathe/carpentry, skilled trade, etc.)
4. General laborer (civil site work, cleaning, janitor, manual labor, etc.)
5. Clerical/technical (company clerical/technical, teacher, etc.)
6. Managerial/administrative position (high-level civil servant above grade 5, corporate manager and above, etc.)
7. Professional/freelance (university professor, lawyer, doctor, religious worker, artist, high-income freelancer, etc.)
8. Homemaker
9. Student (university, graduate student)

9-1. Daycare centre student
9-2. Kindergarten student
9-3. Elementary school student
9-4. Middle school student
9-5. High school student
9-6. Not attending an educational facility

10. Unemployed/retired
11. Other (Please be specific:)

DQ 2) How many people in your household are currently living with you? (including yourself)
* If you live in a 2-person dormitory Monday to Friday and at home with your parents and one sibling on weekends:
* *Note:* A cohabiting member is defined as any individual living in the same household, regardless of blood relationship.
2 people (dormitory) + 4 people (home) - 1 person (to avoid counting yourself twice) = 5 people

Total _________ people

DQ 2-1) You answered that you have (DQ2) people living with you. Please record the ages of all cohabiting members. (record up to a maximum of 10)

①_________-year-old, ②_________-year-old, ③_________-year-old, ④_________-year-old, ⑤_________-year-old

⑥_________-year-old, ⑦_________-year-old, ⑧_________-year-old, ⑨_________-year-old, ⑩_________-year-old

Notice before the survey

※ **Notes before you record**

Please read the notes below carefully.

1. Please complete the information for all people with whom you had social contact during the day.
   ▶ Interactive dialogue consisting of three or more words or skin contact (e.g., a handshake, hug, kiss, or contact sports such as football or basketball)

2. Tips for participating in the survey
   ▶ Record information about every person you had social contact with during the day, regardless of the contact duration or whether you knew the person.
   ▶ If you had multiple contacts with the same person in one day, write only one line per person and include the total contact duration.
   ▶ A day is defined as the period from 00:00 to 24:00 on the specified date.
   ▶ Do not record contact if it was only by mobile or online.

3. What to write
   Enter the following details about each person with whom you had social contact:
   ▶ Age of the contact person
   ▶ Sex of the contact person
   ▶ Social relationship with you
   ▶ Location of contact
   ▶ Residence of the contact person
   ▶ Frequency of contact
   ▶ Duration of contact
   ▶ Size of contact

Explain how to record a information of the social contact

A 1) What is the age of the **first person** you had contact with? If you don't know exactly, please record an estimate.

17-year-old

A 2) What is the sex of the **first person** you had contact with?

1. Male ②. **Female**

A 3) Did you have an interactive dialogue consisting of three or more words, or did you have a skin contact (e.g., a handshake, hug, kiss, or contact sports such as football or basketball) with the **first person** you had contact with? Or was there both an interactive dialogue consisting of three or more words and skin contact (e.g., a handshake, hug, kiss, or contact sports such as football or basketball)?

1. Skin contact (e.g., a handshake, hug, kiss, or contact sports such as football or basketball)
②. Interactive dialogue consisting of three or more words
3. Interactive dialogue consisting of three or more words or skin contact (e.g., a handshake, hug, kiss, or contact sports such as football or basketball)

A 4) Which of the following best describes your social relationship with the **first person** you had contact with?

1. Cohabiting member
2. Extended family (grandparents, nieces, nephews, etc.)
3. Coworker
④. **Educational relationship**
5. Brethren
6. Friend
7. Other (Please be specific:)

A 5) Where did you meet the **first person** you had contact with?
Please select all the places if you had multiple contacts with one person on the same day. (Multiple responses are possible)

1. Home
2. Workplace
③. **Education facility (school, academy)**
4. Religious facility
5. Restaurant/cafe/pub
6. Other (Please be specific:)

A 6) Did the **first person** you had contact with live in the same residence region as you?

①. **Same region** 2. Not the same region→A6-1 3. Unknown

A 6-1) Where did the **first person** you had contact with live?

1. Seoul 2. Busan 3. Deagu 4. Incheon 5. Gwangju 6. Deajeon
7. Ulsan 8. Sejong 9. Gyeonggi 10. Gangwon 11. Chungcheongbuk 12. Chungcheongnam
13. Jeollabuk 14. Jeollanam 15. Gyeongsangbuk 16. Gyeongsangnam 17. Jeju

A 7) How often do you meet with the **first person** you had contact with?

1. everyday
②. **5-6 times a week**
3. 3-4 times a week
4. 1-2 times a week
5. 1-2 times a month
6. less than once a month
7. rarely met

A 8) How much of the day did you spend with the **first person** you had contact with? Please answer the total duration if you had multiple contacts with one person on the same day.
* If you played basketball with a schoolmate for 30 minutes and then drank a cup of coffee in the cafe for 30 minutes:
The total duration is 1 hour (= 30 minutes + 30 minutes).

1. less than 5 minutes
2. 5-15 minutes
3. 15 minutes - 1 hour
④. **1-4 hours**
5. more than 4 hours

A 9) What was the size of contact with the **first person** you had contact with? The size of contact means the total number of people you came into contact with, including yourself. Please answer the total size of contact if you had multiple contacts with one person on the same day.
* If 4 people played basketball with your schoolmates and then 3 people drank a cup of coffee in the cafe:
The total size of the contact is 6 people (= 4 people + 3 people - 1 person(duplicate of yourself)).

6 people

※ Example table entry

MM/DD	1) Age of the contact person *estimate possible	2) Sex of the contact person		3) Type of the contact			4) Relationship with the contact person (select singular)							5) Location of the contact (multiple responses are possible)						6) Whether the contact lives in the same residence region			6-1) Where the contact person lives	7) Frequency of social contact with the contact person (select singular)							8) Duration per day with the contact person (select singular)					9) Contact size (total number of people in social contact)
contact order	hand-written	1. male	2. female	1. only skin contact	2. only dialogue	3. both	1. cohabiting member	2. extended family	3. coworker	4. educational relationship	5. brethren	6. friend	7.other (handwritten)	1. home	2. workplace	3. educational facility	4. religious facility	5. restaurant / cafe / pub	6.other (handwritten)	1. same region	2. not same region	3. unknown	If you know the residence of a contact person. Please write the region (17 si-do)	1. every day	2. 5-6 times a week	3. 3-4 times a week	4. 1-2 times a week	5. 1-2 times a month	6. less than once a month	7. rarely met	1. less than 5 minutes	2. 5-15 minutes	3. 15 minutes - 1 hour	4. 1-4 hours	5. more than 4 hours	Hand-written
1	17	1	②	1	②	3	1	2	3	④	5	6		1	2	③	4	5		①	2	3		1	②	3	4	5	6	7	1	2	3	④	5	6

※ Explain how to record a survey table

‣ The date is indicated in the top left corner. You can record up to 60 contact people per day.

‣ If you had multiple contacts with one person on the same day, please enter only one line per person and write the total duration of the contact.

‣ In (1), record the age of the contact person. If you don't know exactly, record an estimate.

‣ In (2), circle (O) or c (V) the number that corresponds to the sex of the contact person.

‣ In (3), circle (O) or v (V) the number that corresponds to whether the contact had an interactive dialogue consisting of three or more words, a skin contact or an interactive dialogue consisting of three or more words and a skin contact.

‣ In (4), circle (O) or v (V) the number that corresponds to a social relationship with the contact person. The cohabiting member means an individual living in the same house regardless of blood relationship. The extended family is grandparents, nieces and nephews, etc.

‣ In (5), circle (O) or v (V) the number that corresponds to the location where you met the contact person. Select all the locations if you had multiple contacts with one person on the same day.

‣ In (6), circle (O) or v (V) in 1 if the contact person lives in the same residence region as you. If it's not the same region, circle (O) or v (V) in 2 and then record the residence region of the contact person in (6-1).

‣ In (6-1), record the residence region where the contact person lives.
- Below -
1. Seoul 2. Busan 3. Deagu 4. Incheon 5. Gwangju 6. Deajeon 7. Ulsan 8. Sejong 9. Gyeonggi 10. Gangwon 11. Chungcheongbuk 12. Chungcheongnam 13. Jeollabuk 14. Jeollanam 15. Gyeongsangbuk 16. Gyeongsangnam 17. Jeju

‣ In (7), circle (O) or v (V) the number that corresponds to indicate the frequency of contact with the contact person.

‣ In (8), circle (O) or v (V) the number that corresponds to indicate the total duration time of contact you spent with the contact during the day. Please answer the total duration if you had multiple contacts with one person on the same day.

‣ In (9), record the size of contact with the contact person (meaning the total number of contact people, including yourself.). Please answer the total size of the contact if you had multiple contacts with one person on the same day.

MM/DD	1) Age of the contact person *estimate possible	2) Sex of the contact person		3) Type of the contact			4) Relationship with the contact person (select singular)							5) Location of the contact (multiple responses are possible)						6) Whether the contact lives in the same residence region			6-1) Where the contact person lives	7) Frequency of social contact with the contact person (select singular)							8) Duration per day with the contact person (select singular)					9) Contact size (total number of people in social contact)
contact order	hand-written	1. male	2. female	1. only skin contact	2. only dialogue	3. both	1. cohabiting member	2. extended family	3. coworker	4. educational relationship	5. brethren	6. friend	7.other (handwritten)	1. home	2. workplace	3. educational facility	4. religious facility	5. restaurant / cafe / pub	6.other (handwritten)	1. same region	2. not same region	3. unknown	If you know the residence of a contact person. Please write the region (17 si-do)	1. every day	2. 5-6 times a week	3. 3-4 times a week	4. 1-2 times a week	5. 1-2 times a month	6. less than once a month	7. rarely met	1. less than 5 minutes	2. 5-15 minutes	3. 15 minutes - 1 hour	4. 1-4 hours	5. more than 4 hours	Hand-written
1	___	1	2	1	2	3	1	2	3	4	5	6		1	2	3	4	5		1	2	3		1	2	3	4	5	6	7	1	2	3	4	5	___
2	___	1	2	1	2	3	1	2	3	4	5	6		1	2	3	4	5		1	2	3		1	2	3	4	5	6	7	1	2	3	4	5	___
3	___	1	2	1	2	3	1	2	3	4	5	6		1	2	3	4	5		1	2	3		1	2	3	4	5	6	7	1	2	3	4	5	___
4	___	1	2	1	2	3	1	2	3	4	5	6		1	2	3	4	5		1	2	3		1	2	3	4	5	6	7	1	2	3	4	5	___
5	___	1	2	1	2	3	1	2	3	4	5	6		1	2	3	4	5		1	2	3		1	2	3	4	5	6	7	1	2	3	4	5	___
6	___	1	2	1	2	3	1	2	3	4	5	6		1	2	3	4	5		1	2	3		1	2	3	4	5	6	7	1	2	3	4	5	___
7	___	1	2	1	2	3	1	2	3	4	5	6		1	2	3	4	5		1	2	3		1	2	3	4	5	6	7	1	2	3	4	5	___
8	___	1	2	1	2	3	1	2	3	4	5	6		1	2	3	4	5		1	2	3		1	2	3	4	5	6	7	1	2	3	4	5	___
9	___	1	2	1	2	3	1	2	3	4	5	6		1	2	3	4	5		1	2	3		1	2	3	4	5	6	7	1	2	3	4	5	___
10	___	1	2	1	2	3	1	2	3	4	5	6		1	2	3	4	5		1	2	3		1	2	3	4	5	6	7	1	2	3	4	5	___
11	___	1	2	1	2	3	1	2	3	4	5	6		1	2	3	4	5		1	2	3		1	2	3	4	5	6	7	1	2	3	4	5	___
12	___	1	2	1	2	3	1	2	3	4	5	6		1	2	3	4	5		1	2	3		1	2	3	4	5	6	7	1	2	3	4	5	___
13	___	1	2	1	2	3	1	2	3	4	5	6		1	2	3	4	5		1	2	3		1	2	3	4	5	6	7	1	2	3	4	5	___
14	___	1	2	1	2	3	1	2	3	4	5	6		1	2	3	4	5		1	2	3		1	2	3	4	5	6	7	1	2	3	4	5	___
15	___	1	2	1	2	3	1	2	3	4	5	6		1	2	3	4	5		1	2	3		1	2	3	4	5	6	7	1	2	3	4	5	___

◆ Thank you very much. ◆
